# Altered Functional Protein Networks in the Prefrontal Cortex and Amygdala of Victims of Suicide

**DOI:** 10.1371/journal.pone.0050532

**Published:** 2012-12-06

**Authors:** Katalin Adrienna Kékesi, Gábor Juhász, Attila Simor, Péter Gulyássy, Éva Mónika Szegő, Éva Hunyadi-Gulyás, Zsuzsanna Darula, Katalin F. Medzihradszky, Miklós Palkovits, Botond Penke, András Czurkó

**Affiliations:** 1 Laboratory of Proteomics, Institute of Biology, Eötvös Loránd University, Budapest, Hungary; 2 Department of Physiology and Neurobiology, Eötvös Loránd University, Budapest, Hungary; 3 Proteomics Research Group, Institute of Biochemistry, Biological Research Centre, Hungarian Academy of Sciences, Szeged, Hungary; 4 Department of Pharmaceutical Chemistry, School of Pharmacy, University of California San Francisco, San Francisco, Calofornia, United States of America; 5 Neuromorphology Research Group, Hungarian Academy of Sciences, Semmelweis University, Budapest, Hungary; 6 Medical Chemistry Department, University of Szeged, Szeged, Hungary; Mental Health Research Institute of Victoria, Australia

## Abstract

Probing molecular brain mechanisms related to increased suicide risk is an important issue in biological psychiatry research. Gene expression studies on *post mortem* brains indicate extensive changes prior to a successful suicide attempt; however, proteomic studies are scarce. Thus, we performed a DIGE proteomic analysis of *post mortem* tissue samples from the prefrontal cortex and amygdala of suicide victims to identify protein changes and biomarker candidates of suicide. Among our matched spots we found 46 and 16 significant differences in the prefrontal cortex and amygdala, respectively; by using the industry standard *t* test and 1.3 fold change as cut off for significance. Because of the risk of false discoveries (FDR) in these data, we also made FDR adjustment by calculating the *q*-values for all the *t* tests performed and by using 0.06 and 0.4 as alpha thresholds we reduced the number of significant spots to 27 and 9 respectively. From these we identified 59 proteins in the cortex and 11 proteins in the amygdala. These proteins are related to biological functions and structures such as metabolism, the redox system, the cytoskeleton, synaptic function, and proteolysis. Thirteen of these proteins (CBR1, DPYSL2, EFHD2, FKBP4, GFAP, GLUL, HSPA8, NEFL, NEFM, PGAM1, PRDX6, SELENBP1 and VIM,) have already been suggested to be biomarkers of psychiatric disorders at protein or genome level. We also pointed out 9 proteins that changed in both the amygdala and the cortex, and from these, GFAP, INA, NEFL, NEFM and TUBA1 are interacting cytoskeletal proteins that have a functional connection to glutamate, GABA, and serotonin receptors. Moreover, ACTB, CTSD and GFAP displayed opposite changes in the two examined brain structures that might be a suitable characteristic for brain imaging studies. The opposite changes of ACTB, CTSD and GFAP in the two brain structures were validated by western blot analysis.

## Introduction

Suicide is a human attribute without a proper equivalent in animals; however, some behavioural traits, such as aggression, hopelessness, and impulsivity, are correlated with suicide and can be reproduced in animals [1]. Suicidal behaviour often occurs in conjunction with different psychiatric diseases, such as major depression or schizophrenia [2]. Major depression and bipolar disorder generally increase the incidence of suicide [3].

Although suicide is a complex behaviour that is often preceded by suicidal thoughts, it can occur as the outcome of an impulsive action [4]. The altered serotonergic transmission theory is the most widely emphasised cellular mechanism of suicide [4], [5]. Suicide is linked with the downregulation of serotonin (5HT) release and/or uptake [6] together with 5-HT1A receptor dysfunction. These dysfunctions are thought to be major factors in several mental disorders, including major depression [7]; however, the current gene expression data suggest that suicide is possibly correlated with extensive changes in the brain and is not restricted to only one neurotransmitter system [8], [9], [10]. In addition to changes that have been observed in the serotonergic system, studies on brain samples of people who have committed suicide suggest that GABAergic and glutamatergic transmissions are also involved [11], [12]. Furthermore, changes in the expression of glia-derived genes and glial fibrillary acidic protein (GFAP) in depression and other psychiatric illnesses indicate that suicide-related molecular alterations may not be restricted to neurons [13]. Most likely, molecular mechanisms in the brain that lead to suicide coexist with pathological changes along several functional protein networks. Suicide-brain studies that show that hyper-methylation of the ribosomal-RNA gene promoter could cause aberrant changes in protein synthesis [14] support this idea. Psychoactive drugs can change the risk of suicide, and there are ongoing efforts to find potential biomarkers to predict suicidal behaviours [15], [16], [17], [18], [19], [20]. Thus, understanding the molecular brain mechanisms involved in suicide is important for the development of both psychoactive drugs and predictive diagnostic tools.

Screening technology progress in the past two decades (e.g., the gene chip and the 2D gel-based and liquid-based proteomic techniques) have provided new insights into the molecular processes of the brain [21]. Because suicide cannot be observed in animals, investigating *post mortem* human brains with a relatively short *post mortem* delay is a good alternative. Particularly, the *post mortem* human brain proteome reflects the complex pathological changes of protein expression in the human brain while alive [21]. A homogeneous sample is usually unlikely in such studies because suicide and its associated psychiatric disorders and medications differentially influence various underlying molecular mechanisms. Therefore, in the present study we used brain samples from people who had hanged themselves and from individuals who died due to acute cardiac arrest to decrease the heterogeneity of data. We examined prefrontal cortex and amygdala samples because mood disorders invoke several neuronal mechanisms in these brain areas and are correlated with suicide [1], [7].

Our aim was to find changes in the proteome of the prefrontal cortex and amygdala that correlated with suicide. Changes in protein expression patterns may reflect molecular changes of psychopathological states and could provide biomarkers for suicide risk.

## Methods

### Ethics Statement

The human brains were obtained from the Lenhossek Human Brain Program, Human Brain Tissue Bank, Budapest. Brains were taken from persons who had died without any known neurodegenerative diseases. The collection of brains and the microdissection of the brain samples for research have been performed by the approval of the Regional Committee of Science and Research Ethics of the Semmelweis University, Budapest (TUKEB: 32/92) and the Ethics Committee of the Ministry of Health, Hungary, 2002 according to the principles expressed in the Declaration of Helsinki. Tissues were collected only after a family member gave informed (written) consent.

### Sample Collection and Preparation for Proteomics

We used brain samples from male subjects. The age distributions of suicide (6 brains; age range: 41–79 years; mean age: 52.7; SD: 14.2) and control (6 brains; age range: 47–85 years; mean age average: 64.8; SD: 17.2) groups did not differ significantly (p = 0.1481, Wilcoxon test; Table 1). Suicide group brain samples came from subjects who had hanged themselves, control group brain samples came from victims of cardiac arrest. No information was available whether the cardiac arrest in control subjects happened during sleep or not. The *post mortem* interval (PMI) did not differ significantly between groups (p = 0.0683, Wilcoxon test; Table 1). We used two brain areas - the prefrontal cortex and the amygdala – to conduct proteomic analyses. We treated and handled brain samples as described in a previous publication [22]; briefly, brains were removed from the skull 2–6 hours after death, frozen, and sliced into 1– to 1.5 cm-thick coronal sections. We used the punch technique to micro-dissect the brain areas. Tissue samples were stored at –80°C until used. In this study, we processed one cortex and one amygdala samples from 6 suicide and 6 control subjects, meaning a total of 24 human *post mortem* brain tissue samples.

**Table 1:** Description of participants in the present study.

Brain No.	Gender	Age	*Post mortem* interval (PMI)	Cause of death	Neuropathological diagnosis
**#138 S**	male	52	3 h	suicide (hanging)	lack of specific neuropathological alteration
**#139 S**	male	79	4 h	suicide (hanging)	lack of specific neuropathological alteration
**#143 S**	male	43	3 h	suicide (hanging)	lack of specific neuropathological alteration
**#144 S**	male	42	4 h	suicide (hanging)	lack of specific neuropathological alteration
**#173S**	male	43	6 h	suicide (hanging)	NA
**#174S**	male	57	6 h	suicide (hanging)	NA
**#11 C**	male	47	2 h	acute cardiac insufficiency, chronic myocardial infarction, chronic heart failure, coronary sclerosis	NA
**#12 C**	male	80	2 h	acute cardiac insufficiency, acute heart failure, coronary sclerosis, senile, hypertensive arteriosclerosis	NA
**#111 C**	male	55	3 h	cardiac insufficiency, coronary stenosis	NA
**#151 C**	male	47	2 h	acute myocardial infarction	encephalopathia alcoholica
**#164 C**	male	85	3 h	cardiorespiratory insufficiency	lacunar encephalopathy
**#213 C**	male	75	5 h	cardiac insufficiency	vascular leucoencephalopathysmall vessels diseaselacunar stroke

NA: not available; S: suicide; C: control.

The brain sample preparation protocol was similar to previous studies [23], [24]; briefly, we mechanically homogenised tissue samples in a cooled lysis buffer (7 M urea; 2 M thiourea; 20 mM Tris; 5 mM magnesium acetate, 4% CHAPS; Protease Inhibitor Mix (1∶1000), GE Healthcare, Uppsala, Sweden). Samples were then sonicated and centrifuged (1 h, 14000 *g*, 4°C). The pH of the supernatant was adjusted to 8.0 and protein concentrations of the samples were measured by PlusOne Quant Kit (GE Healthcare). We labelled 5 µg of each protein sample with CyDye™ DIGE Fluor Labelling kit for Scarce Samples (GE Healthcare) at a concentration of 4 nmol/5 µg proteins according to instructions.

We labelled the experimental samples (control and suicide samples) as Cy5 and the pooled internal standard samples (reference or standard sample, is a pool comprising equal amounts (2.5 µg) of each of the experimental samples being compared) as Cy3. The pooled standard represents the average of all the samples being analyzed and ensures all proteins present in the experimental samples are represented. The pooled standard is used to normalize protein abundance measurements across multiple gels in an experiment. As a consequence each gel will contain an image with a highly similar spot pattern, simplifying and improving the confidence of inter-gel spot matching and quantification [25].

We multiplexed the differently labelled samples in the same gel. Sample multiplexing in DIGE greatly refines the detection of changes at the protein level between samples [26], as variation in spot intensities due to experimental factors, for example protein loss during sample entry into the strip, will be the same for both samples within a single DIGE gel [25].

The multiplexed, differently labelled samples (5 µg protein of Cy5-labelled and 5 µg protein of Cy3-labelled reference) were dissolved in isoelectric focusing (IEF) buffer containing ampholytes (0.5 v/v %), DTT (0.5 m/v %), 8 M urea, 30% glycerine, 2% CHAPS, and rehydrated passively onto 24 cm nonlinear IPG strips (pH 3–10 NL, GE Healthcare) overnight at room temperature. After rehydration, the strips were placed to first dimension isoelectric focusing (IPGPhore, GE Healthcare) for 24 h to attain a total of 80 kVh. The applied currents were: 30 V for 3.5 h step, 500 V for 5 h gradient, 1000 V for 6 h gradient, 8000 V for 3 h gradient, and 8000 V for 6.5 h step mode. Focused proteins were reduced by equilibrating with buffer containing 1% (w/v) mercaptoethanol for 20 min. After reduction the IPG strips were loaded onto 10% polyacrylamide gels (24×20 cm), and SDS-PAGE was conducted at 2 W/gel for 1 h and at 10 W/gel in the second dimension.

We prepared 12 gels from both areas because one experimental sample and one pooled standard reference sample can be loaded into one gel with the Labelling kit for Scarce Samples (GE Healthcare). Following electrophoresis, gels were scanned by a Typhoon TRIO+ Variable Mode Imager (GE Healthcare) using appropriate lasers and filters with the photomultiplier tube (PMT) biased at 600 V. Cy3 images were scanned using a 532 nm laser and an emission filter of 580 nm BP (band pass) 30. Cy5 images were scanned using a 633 nm laser and a 670 nm BP30 emission filter. All gels were scanned at 100 µm resolution. Images in different channels were overlaid using selected colours, and differences were visualised using Image Quant software (GE Healthcare). We used the DeCyder 6.5 2D gel evaluation software (GE Healthcare); the Differential In-gel Analysis (DIA) module to perform differential protein analyses and the Biological Variance Analysis (BVA) module to gel-to-gel matching and statistical analysis of protein-abundance change between samples.

In the DIA module the scanned images of the sample and the internal standard were overlaid and the algorithms within the software co-detected the spots in the gel. The estimated number of spots for each co-detection procedure was set to 2500. When calculating the abundance ratios for spot pairs in co-detected sample images, the spot volumes of the component spot maps needed to be normalized and the log standardized abundances were calculated.

The statistical analysis of protein-abundance change between samples was made by the BVA module. The BVA matched the quantified spots of all gels to a chosen master gel. According to the standard proteomic protocol [25], the threshold for the differential expression was set at a minimum fold change of 1.3 as we used human samples and the quality of the gels were adequate. We determined the p-values (Student’s *t*-test) for each protein spot (p<0.05).

To identify proteins in the spots of interest, we performed preparative 2D electrophoresis using 800 µg of proteins per gel. We made four preparative gels and picked the relevant spots for protein identification.

### Protein Identification

We extracted peptides from gel spots after in-gel digestion by Trypsin Gold (for a detailed protocol, see http://ms-facility.ucsf.edu/ingel.html). Peptide separation before MS analysis was done by HPLC started by inline trapping on to a nanoACQUITY UPLC trapping column (Symmetry, C18 5 µm, 180 µm × 20 mm; 15 µl/min with 3% solvent B) followed by a linear gradient elution (solvent B: 10% to 50% in 40 min, flow rate: 250 nl/min; nanoACQUITY UPLC BEH C18 Column, 1.7 µm, 75 µm × 200 mm). Solvent A was composed of 0.1% formic acid in water; solvent B was composed of 0.1% formic acid in acetonitrile. MS measurements started by using information-dependent acquisition mode, using a Waters nanoAcquity nanoUPLC system coupled to a Micromass qTOF tandem mass spectrometer (Waters, USA). Next, 3 s collision-induced dissociation (CID) analyses on multiple computer-selected ions were performed for amino acid sequence determination.

### Database Search

We converted raw MS data into a Mascot generic file using the Mascot Distiller software (version 2.1.1.0). We used the Mascot search engine (version 2.2.2) to search the resulting peak lists against the NCBI non-redundant database without species restriction (6,833,826 sequences), to eliminate false positive hits. We submitted monoisotopic masses with a peptide mass tolerance of at least 50 ppm and a fragment mass tolerance of at least 0.1 Da. We set the carbamidomethylation of Cys as a fixed modification, and we permitted acetylation of the protein N-termini, methionine oxidation and pyroglutamic acid formation from N-terminal Gln residues as variable modifications. The acceptance criterion was the identification of at least two significant peptides per protein (i.e., peptide score >52, p<0.05).

### Correction for False Discovery Rate (FDR)

When applying statistical tests to 2-D gel data, one is faced with the so-called multiple hypothesis testing problem: for each matched and quantified spot series, a separate test is done. Each test has a certain probability of giving a false positive result, and the large number of tests can produce a high number of false positives [27]. This has led to the application of methodologies to control the false discovery rate (FDR) where FDR is the rate of false positive results among all profiles that were tested positive (type I errors).

The original FDR methodology was considered to be too conservative for discovery experiments consequently, an extension to the FDR was developed by Storey that calculates a *q-*value [28].

The *q-*values were calculated from the *p-*values obtained for all features within the study with the statistics software, R (R Development Core Team (2011)). R: A language and environment for statistical computing (R Foundation for Statistical Computing, Vienna, Austria. ISBN 3-900051-07-0, URL http://www.R-project.org/) [29] by using an easy to use tool (QVALUE software ver. 1.0) developed by Storey and Tibshirani [28]. The frequency distributions of *P*-values were used to estimate the proportion of features that are unchanging; this is then used to estimate the false discovery rate (Fig. S1).

Careful observation of the *P*-values histograms suggested that the shape of the histograms were not the most desirable shape, although they were acceptable. Note, that the Student’s *t* test we used is a simple test that assumes the data are randomly sampled from normal distributions and shows homogeneity of variance. In DIGE with the traditional three-dye approach, Karp et al. demonstrated that the final standardized abundance data for the spots are not truly independent [30]. However, we used the two-dye design in this study where the Student’s *t* test was adequate [30], [31].

The histograms of *P*-values of the prefrontal cortex and amygdala were dense near zero and became less dense as the *P*-values increased. The amygdala *P*-histogram contained a wider peak indicating that less spots were detected as significantly changing. By observing their *q-*value cut-off histograms (Fig. S1) we used 0.06 and 0.4 as *q-*values alpha thresholds for FDR adjustment of significant spots of the prefrontal cortex and amygdala, respectively.

### Functional Clustering of Identified Proteins

Following an extensive literature search, we formed the functional protein clusters using PDB (http://www.pdb.org, La Jolla, CA, USA), ExPASy and UniProt databases (http://www.expasy.org and http://www.uniprot.org, respectively; Swiss Institute of Bioinformatics, Switzerland). From our data pool we selected 11 proteins that changed in both the amygdala and the cortex for detailed protein interaction modelling analyses using PathwayStudio® 6.2 software (Ariadne Genomics, Inc., Rockville, MD, USA). The protein network model created was manually verified using the PubMed database (http://www.ncbi.nlm.nih.gov, MD, USA).

### Western Blot

Frozen brain samples were homogenized as described earlier [24]. Protein lysates (20 µg) were resolved on a 10% polyacrylamide gel. Proteins were transferred onto a nitrocellulose membrane (Bio-Rad, USA). Membranes were blocked in 5% BSA in TRIS-Tween buffer (500 mM TRIS, 150 mM sodium chloride, pH 7.4, and 0.05% Tween 20 (Sigma)) for 1 h, incubated with polyclonal anti-cathepsin 1B (1∶1000, Santa Cruz Biotechnology, CA, USA), anti-GFAP (1∶1000, DAKO, Denmark) or anti-actin (1∶5000, Sigma, Hungary) antibodies in TRIS-Tween buffer for 24 h at 4°C. After incubation with ECL-HRP-conjugated secondary antibody (1∶5000, GE Healthcare, Germany), bands were visualized using a Chemiluminescence kit (BioRad, CA, USA). Ponceau staining was used as control for equal protein load and transfer.

## Results

**Figure 1:**
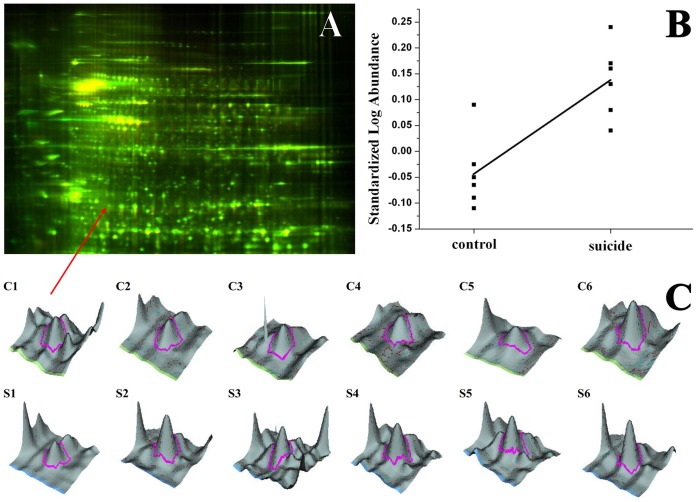
Representative gel image. The first dimension was carried out in pH 3–10 NL IPG strip and the second dimension was 24×20 cm 10% SDS PAGE. Part A shows the overlaid image, part B shows the standardized log abundance of a representative spot (2406, prefrontal cortex) on the different gels, part C shows 3D views of the individual spots (C1–C6: control brains; S1–S6: suicide brains).

We used DIGE proteomics technology to investigate the differences in the protein expression pattern of suicide compared to control brain samples. We detected a total of 2,465 spots (after exclusion of false spots) from the prefrontal cortex and 2,115 from the amygdala on the master gels, defined to be the gel containing the most spots. Representative gel is shown in Figure 1. We performed the spot gel-to-gel matching with the DeCyder 6.5 software (GE Healthcare) BVA module and after careful and rigours manual validation we matched 681 spots in the prefrontal cortex samples and 696 in the amygdala samples. From these matched spots with the *t* test and 1.3 fold change as cut off we found 46 significant differences between the control and suicide prefrontal cortex samples from which we could identify 84 proteins (see Table S1). Regarding the amygdala, 16 matched spots showed significant differences, and 20 proteins were identified from them (see Table S2). After FDR adjustment we had 27 significant spots in the prefrontal cortex and 9 significant spots in the amygdala. This way the number of protein “hits” in the proposed profile reduced to 59 proteins in the prefrontal cortex and 11 proteins in the amygdala (see bold-italic gene names in Tables 2 and 3).

**Table 2:** Functionally clustered protein changes in the prefrontal cortex.

CYTOSKELETON					
Gene	Protein name	Up/down regulation	Accessionnumber	Cellular localization	Molecular function
* *+ACTB*[82]	Actin, cytoplasmic 1	↓	P60709	Cytoplasm, cytoskeleton	Structural constituent of cytoskeleton, cell motion
* *INA*[83], [84], [85], [86]	Alpha-internexin	↑	Q16352	Neurofilament	Cell differentation, nervous system development, structural constituent of cytoskeleton
*+*NEFL*[87], [66], [88], [89]	Neurofilament, light polypeptide 68kDa	↑↑↓	P07196	Axon, neurofilament	Maintenance of neuronal caliber, axon cargo transport
* *NEFM*[69]	Neurofilament, medium polypeptide	↑↑↑↑	Q4QRK6	Axon, intermediate filament, neurofilament, neuromuscular junction	Axon cargo transport, microtubule/neurofilament cytoskeleton organization, regulation of axon diameter
*SERPINB3*	Serpin B3	↓	P29508	Cytoplasm	Protein binding, serine-type endopeptidase inhibitor activity
* *TUBA1A*[90]	Tubulin alpha-1B chain	↑↑↓	P68363	Microtubule	Microtubule-based movement, protein polymerization
*TUBA1B[90]	Tubulin alpha-1C chain	↑	Q9BQE3	Microtubule	Major constituent of microtubules, microtubule-based movement, protein polymerization
* *TUBA1C*[90]	Tubulin alpha-4A chain	↑↑↓	P68366	Cytoplasm, microtubule	Microtubule-based movement, protein polymerization, major constituent of microtubules
TUBB4	Tubulin beta-4 chain	↓	P04350	Cytoplasm, microtubule	Major constituent of microtubules, microtubule-based movement, protein polymerization

SIGNALING					
Gene	Protein name	Up/down regulation	Accession number	Cellular localization	Molecular function
* *CALB2*[91], [92], [93]	Calbindin 2	↓	P22676	N.D.	Calcium ion binding
*CAP2*	Adenylyl cyclase-associated protein 2	↑	P40123	Cell membrane	Signal transduction, establisment/maintenance of cell polarity, cytoskeleton organization, activation of adenylate cyclase activity
*CRKL*	Crk-like protein	↑	P46109	Cytoplasm	JNK cascade, Ras protein signal transduction, protein tyrosine kinase activity
* *EFHD2*[69]	EF-hand domain family, member D2	↓↓	Q96C19	Membrane raft	Calcium ion binding, regulator of the NF-kappa-B-activating branch, apoptosis
+*GRB2*[94]	Growth factor receptor-bound protein 2	↓	P62993	Golgi apparatus, cytosol	Ras protein signal transduction, cell-cell signaling, interspecies interaction between organisms, insulin receptor signaling pathway, EGFR signaling pathway
S *+ *MAPK3*[95], [96], [97]	Mitogen-activated protein kinase 3 (ERK1)	↑	P27361	Cytoplasm, cytoskeleton, nucleoplasm	Cell cycle, Ras protein signal transduction, protein amino acid phosphorilation, interspecies interaction between organisms
PARK7	Protein DJ-1	↓	Q99497	Cytoplasm, nucleus	Chaperone, Ras protein signal transduction
*PGK1*	Phosphoglycerate kinase 1Serine/threonine-protein phosphatase 2B catalytic subunit alpha isoform	↑↑↑↑	Q08209	Cytosol, nucleus	Calcium ion binding, calmodulin binding, iron ion binding, zinc ion binding
YWHAB	Tyrosine 3-monooxygenase/tryptophan 5-monooxygenase activation protein, beta polypeptide14-3-3 protein beta/alpha	↑	P31946	Cytoplasm, melanosome	Signal transduction, regulation of amino acid dephosphorilation, apoptosis
*YWHAG*	14-3-3 protein gamma	↓	P61981	Cytoplasm	Signal transduction, synaptic plasticity, neuron differentation, regulation of protein kinase activity
S *YWHAE[74], [98]	14-3-3 protein epsilon	↑	P62258	Cytosol, melanosome	Apoptosis, intracellular signaling cascade, interspecies interaction betweenorganisms
*+YWHAH[72], [99]	14-3-3 protein eta	↑	Q04917	Cytoplasm	Glucocorticoid catabolism/signaling, synaptic plasticity, dendrite morphogenesis, regulation of transcription, protein transport
*YWHAZ[88], [89], [69],[73]	14-3-3 protein zeta/delta	↑	P63104	Cytoplasm, melanosome	Anti-apoptosis, signal transduction

METABOLISM					
Gene	Protein name	Up/down regulation	Accession number	Cellular localization	Molecular function
ABHD14B	Abhydrolase domain-containing protein 14B	↓	Q96IU4	Cytoplasm, nucleus	Hydrolase activity
*ALDOC[85], [90], [68], [100], [101], [102]	Fructose-bisphosphate aldolase C	↑	P09972	Cytoskeleton	Glycolysis, fructose 1,6-bisphosphate catabolism
* *APOA1*[103]	Apolipoprotein A-I	↑	P02647	Endocytic vesicle, ER lumen, plasma membrane, secretory granule	Cholesterol metabolism, lipid metabolism/transport, sterod metabolism, transport
*ATP5B[104]	ATP synthase subunit beta, mitochondrial	↓	Q13510	Membrane, mitochondria	ATP synthesis, hydrogen ion transport, ion transport, transport, angiogenesis, regulation of intracellular pH
*ATP5C1*	ATP synthase, H+ transporting, mitochondrial F1 complex, gamma polypeptide 1	↓	P06576	Mitochondria	ATP synthesis, hydrogen ion transport, ion transport, transport
ATP5H	ATP synthase, H+ transporting, mitochondrial Fo complex, subunit d	↓	O75947	Mitochondria	Mitochondrial ATP synthesis coupled proton transport, hydrogen ion transport,
ATP6V1D	ATPase, H+ transporting, lysosomal 34kDa, V1 subunit D	↑	P21281	N.D.	ATP synthesis, hydrogen ion transport, ion transport, transport
*C5orf33*	Chromosome 5 open reading frame 33	↑	Q4G0N4	N.D.	Metabolic process, NAD+ kinase activity
* *CBR1*[69]	Carbonyl reductase [NADPH] 1	↑↑↓	P16152	Cytoplasm	Drug metabolism, vitamin K metabolism
*+*CKB*[85], [88], [90], [105], [100], [106]	Creatine kinase B-type	↑↓↓	P12277	Cytoplasm	Creatine metabolism
*+*CS*[104], [107], [108]	Citrate synthase, mitochondrial	↑↑↑	O75390	Mitochondria	Cellular carbohydrate metabolism, tricarboxylic acid cycle
*ECHS1*	Enoyl-CoA hydratase, mitochondrial	↓↓	P30084	Mitochondria	Fatty acid metabolism, lipid metabolism
*FH*	Fumarate hydratase, mitochondria	↑	P07954	Mitochondria	Fumarate metabolism, tricarboxylic acid cycle
GLOD4	Glyoxalase domain-containing protein 4	↑	Q9HC38	Mitochondria	N.D.
*+*GLUL*[69], [90], [102], [109], [110],	Glutamate-ammonia ligase, glutamine synthetase	↑↑	P15104	Cytoplasm, Golgi apparatus	Cell proliferation, glutamine biosynthesis
*GOT1*	Glutamic-oxaloacetic transaminase 1, soluble (aspartate aminotransferase 1)	↑↑	P17174	Cytoplasm	Aspartate catabolism, cellular response to insulin stimulus, response to glucocorticoid stimulus
GUK1	Guanylate kinase	↓	Q16774	Cytosol	Purine nucleotid metabolism
HADH	Hydroxyacyl-CoA dehydrogenase, mitochondrial	↑	Q16836	Mitochondria	Fatty acid metabolism, lipid metabolism
*IDH2*	Isocitrate dehydrogenase 2 [NADP+], mitochondrial	↑↑	P48735	Mitochondria	Isocytrate metabolism, tricarboxylic acid metabolism, glyoxylate cycle
*IDH3A*	Isocitrate dehydrogenase 3 [NAD+] subunit alpha, mitochondrial	↑	P50213	Mitochondria	Carbohydrate metabolism, tricarboxylic acid cycle
*+IMPA1[111], [112]	Inositol(myo)-1(or 4)-monophosphatase 1	↓	P29218	Cytoplasm	Phosphate metabolism, phosphatidylinositol biosynthesis, signal transduction
* *NDUFS1*[113]	NADH dehydrogenase (ubiquinone) Fe-S protein 1, 75kDa (NADH-coenzyme Q reductase)	↓	P28331	Mitochondrial inner membrane space, respiratory chain complex I	ATP metabolism, transport, electron transport, ROS metabolism, apoptosis
NDUFV2	NADH dehydrogenase FeS protein	↓	Q6IPW4	Mitochondria	NAD binding
+*PDHA1*[114]	Pyruvate dehydrogenase (lipoamide) alpha 1	↑↑↑	O00330	Mitochondria	Pyruvate metabolism
*PDIA3*	Protein disulfide isomerase family A, member 3 Phosphoglycerate kinase 1	↑↑	P00558	Cytoplasm	Glycolysis, phosphorilation
* *PGAM1*[69], [90], [102]	Phosphoglycerate mutase 1	↓↓	P18669	Cytosol	Respiratory burst, glycolysis, pentose-phosphate shunt
*PGLS*	6-Phosphogluconolactonase	↓↓	O95336	Cytoplasm	Pentose-phosphate shunt
*PKM2*	Pyruvate kinase isozymes M1/M2	↑↑	P14618	Cytoplasm, nucleus	Glycolysis, programmed cell death
*TALDO1*	Transaldolase 1	↑	P37837	Cytoplasm	Pentose shunt
*UCHL1*	Ubiquitin carboxyl-terminal esterase L1 (ubiquitin thiolesterase)Pyruvate dehydrogenase E1 component subunit alpha, somatic form, mitochondrial	↑↑	P08559	Mitochondria	Glycolysis, pyruvate metabolism
*UQCRC2*	Ubiquinol-cytochrome c reductase core protein II Cytochrome b-c1 complex subunit 2, mitochondrial	↑↑↑↑	P22695	Mitochondria	Electron transport, respiratory chain, proteolysis, transport, oxidative phosphorylation

PROTEIN PROCESSING					
Gene	Protein name	Up/down regulation	Accession number	Cellular localization	Molecular function
BRCC3	BRCA1/BRCA2-containing complex, subunit 3Lys-63-specific deubiquitinase BRCC36	↑	P46736	Nucleus	DNA repair, modification-dependent protein catabolism, Ubl conjugation pathway
CAPZA2	Capping protein (actin filament) muscle Z-line, alpha 2	↑		Cytoplasm	Chaperon protein folding
+*FKBP4*[115], [71]	FK506-binding protein 4, 59kDa	↑	Q02790	Cytoplasm, nucleus	Protein binding, HSP binding, FK506 binding, peptidyl-prolyl cis-trans isomerase activity
*+*HSPA8*[69], [116]	Heat shock cognate 71 kDa protein	↑↓	P11142	Cytoplasm	Chaperone, response to unfolded proteins, membrane organization, interspecies interaction between organisms, post-Golgi vesicle-mediated transport
* *HSPB1*[117]	Heat shock 27kDa protein 1 (beta-1)	↓	P04792	Cytoplasm, nucleus, cytoskeleton	Anti-apoptosis, cell death, cell motion, response to heat, response to unfolded proteins, regulation of translational initiation
*VTA1*	Vps20-associated 1 homolog	↑	Q9NP79	Cytoplasm, endosome, cell membrane	Protein transport

DEVELOPMENT					
Gene	Protein name	Up/down regulation	Accession number	Cellular localization	Molecular function
*+*DPYSL2*[88], [100], [101], [70], [118], [119]	Dihydropyrimidinase-like 2	↑↑↑	Q16555	Cytolplasm	Cell differentation, nervous system development, nucleotide and nucleic acid metabolism, intracellular trafficking of heterooligomeric forms of steroid hormone receptors
*+*SELENBP1*[120], [67], [121]	Selenium binding protein 1	↑↑	Q13228	Cytoplasm, membrane, nucleus	Protein transport, transport, selenium binding
SEPT2	Septin-2	↑	Q15019	Cytoplasm, cytoskeleton, nucleus	Cell division, mitosis, cell cycle
* *SEPT3*[86], [90]	Neuronal-specific septin-3	↑↑	Q9UH03	Synapse, nucleus, cell junction	Cell cycle, cytokinesis
*+*SEPT5*[119], [122]	Septin 5	↑↑	Q99648	Plasma membrane, septin complex, synaptic vesicle	Cell cycle, regulation of exocytosis
*SIRT2*	NAD-dependent deacetylase sirtuin-2	↑	Q8IXJ6	Cytoplasm, microtubule	Regulation of mitosis, regulation of phosphorilation, chromatin silencing, cell division
*TPD52*	Tumor protein D52	↓	P55327	Endoplasmic reticulum, cytosol	B cell differentation, secretion, anatomical structure morphogenesis
TPD52L2	Tumor protein D52-like 2	↓	O43399	Cytoplasm	Regulation of cell proliferation

SYNAPSE, NEURON					
Gene	Protein name	Up/down regulation	Accession number	Cellular localization	Molecular function
*APOA1BP*	Apolipoprotein A-I-binding protein	↑	Q6PGN4	N.D.	
* *APOL2*[123]	Apolipoprotein L2	↑	Q9BQE5	Cytoplasm	Lipid transport, lipoprotein metabolism, acut-phase response, multicellular orgaismal development
*+*ATP6V1B2*[104], [124]	V-type proton ATPase subunit B, brain isoform	↑↑↑↓	P36542	Plasma membrane, Golgi apparatus, cytosol, melanosome	Hydrogen ion transport, ion transport, transport
*SYN1*	Synapsin-1	↑	P17600	Synaptic vesicles	Neuronal phosphoprotein that coats synaptic vesicles, neurotransmitter release regulatiom
*VDAC1*	Voltage-dependent anion channel 1	↑↑↓↓	P21796	Mitochondria outer membrane, cell membrane	Apoptosis, host-virus interaction, ion transport, transport
*VDAC2*	Voltage-dependent anion channel 2	↑↑	P45880	Mitochondria outer membrane	Ion transport, transport

PROTEOLYSIS					
Gene	Protein name	Up/down regulation	Accession number	Cellular localization	Molecular function
S CAPNS1[125]	Calpain small subunit 1	↓	P04632	Cytoplasm, cell membrane, nucleus	Regulation of cell proliferation
S * *CTSD*[125], [126],	Cathepsin D	↓↓↓	P07339	Lysosome, melanosome, extracellular region	Cell death, proteolysis
+PSMB4[127]	Proteasome subunit beta type-4	↓	P28070	Centrosome, nucleus, proteasome core complex	Regulation of ubiquitin-protein ligase activity during mitotic cell cycle, ubiquitin-dependent protein catabolism

RNA/DNA METABOLISM					
Gene	Protein name	Up/down regulation	Accession number	Cellular localization	Molecular function
*HNRPDL*	Heterogeneous nuclear ribonucleoprotein D-like	↑	O14979	Cytoplasm, heterogenous nuclear ribonuclear complex	Regulation of transcription, RNA processing, transcription
*PHB*	Prohibitin	↓	P35232	Mitochondria, nucleoplasm	DNA replication, cell proliferation, transcription, apoptosis, signal transduction
+*PURA*[128]	Transcriptional activator protein Pur-alpha	↑	Q00577	Nucleus	DNA replication initiation, transcription

REDOX SYSTEM					
Gene	Protein name	Up/down regulation	Accession number	Cellular localization	Molecular function
*GSTM3*	Glutathione S-transferase Mu 3 (brain)	↑↑	P21266	Plasma membrane	Oxidative stress, prevention of cellular degeneration
*+GPX1[129], [130]	Glutathione peroxidase	↓	P07203	Cytosol, mitochondrion	UV-protection, anti-apoptosis, cell redox homeostasis, glutathione metabolism, oxidation reduction, hydrogen peroxide catabolism, regulation of caspase activity
PDHX	Pyruvate dehydrogenase complex, component X Protein disulfide-isomerase A3	↑	P30101	Endoplasmatic reticulum lumen, melanosome	Cell redox homeostasis, protein import into nucleus, signal transduction, protein retention into ER lumen
*+*PRDX6*[68], [131]	Peroxiredoxin 6	↓	P30041	Cytoplasm, lysosome, cytoplasmic vesicle, nucleus	Cell redox homeostasis, oxidation reduction, phospholipid catabolism

GLIA CELLS					
Gene	Protein name	Up/down regulation	Accession number	Cellular localization	Molecular function
S *+*GFAP*[86], [88], [69], [101], [119], [13], [132], [133], [134],	Glial fibrillary acidic protein isoform 1	↑↓↓↓↓↓↓↓↓↓	P14136	Cytoplasm, intermedier filament	Central nervous system development, structural constituent of cytoskeleton

* : proteins involved in schizophrenia; +: proteins involved in depression; S: proteins involved in suicide.

* : proteins involved in schizophrenia; +: proteins involved in depression; S: proteins involved in suicide. Bold-italic gene names highlighting those proteins that were found in those differently expressed protein spots that proved significant with both statistical tests.

↑ or ↓: the direction of the spot intensity change of a given spot compared to control.

Clustering of proteins in the prefrontal cortex revealed the following categories: cytoskeleton, signalling, metabolism, protein processing, development, synapse and neuron, proteolysis, RNA/DNA metabolism, redox system, and glia cell marker (see Table 2). Changes in the protein expression pattern of the amygdala were smaller, but they formed almost the same clusters as the cortical protein changes (see Table 3). The direction of change in the two brain structures was the opposite for several proteins. We identified several proteins in more than one spot of the 2D gel, most likely due to posttranslational or *post mortem* processing. Thus, whenever more than one arrow is included, they represent the number of spots in which the protein was identified; the direction of each arrow shows the direction of change in a certain spot (see Tables 2 and 3). The numerical values of changes and *p*-values of significance are shown in the Supplementary material (see Table S1 and S2).

**Table 3:** Functionally clustered changes in proteins of the amygdala.

CYTOSKELETON					
Gene	Protein name	Up/down regulation	Accession number	Cellular localization	Molecular function
* *+ACTB* [82]	Actin, cytoplasmic 1	↑	P60709	Cytoplasm, cytoskeleton	Structural constituent of cytoskeleton, cell motion
* *INA* [83], [84], [85], [86]	Alpha-internexin (66 kDa neurofilament protein)	↑	Q16352	Neurofilament	Cell differentation, nervous system development, structural constituent of cytoskeleton
*+*NEFL* [87], [66], [88], [89]	Neurofilament, light polypeptide 68 kDa	↑↑↑↑↓	P07196	Axon, neurofilament	Maintenance of neuronal caliber, axon cargo transport
* *NEFM* [69]	Neurofilament, medium polypeptide	↑	Q4QRK6	Axon, intermedier filament, neuromuscular junction	Cytoskeleton organization, axon cargo transport
* *TUBA1A* [90]	Tubulin alpha-1A chain	↑↑	Q71U36	Cytosol, melanosome	Microtubule-based movement, protein polymerization
+TUBB3[135]	Tubulin beta-3 chain	↑	Q13509	Microtubule	Microtubule-based movement, protein polymerization
*+*VIM* [69], [105], [136]	Vimentin	↑	P08670	Cytosol, intermedier filament	Cell motion, structural constituent of cytoskeleton

SIGNALLING					
Gene	Protein name	Up/down regulation	Accession number	Cellular localization	Molecular function
*ANXA5[137], [138]	Annexin A5	↑	P08758	Cytoplasm	Anti-apoptosis, coagulation, signal transduction
*EFHD2[69]	EF-hand domain family, member D2	↑	Q96C19	Membrane raft	Calcium ion binding, regulator of the NF-kappa-B-activating branch, apoptosis
*+PHB[105], [139], [131]	Prohibitin	↑	P35232	Membrane, mitochondria	Proliferation, transcription, apoptosis, replication, signal transduction

METABOLISM					
Gene	Protein name	Up/down regulation	Accession number	Cellular localization	Molecular function
*ATP5A1*	ATP synthase subunit alpha, mitochondrial (precursor)	↓	P25705	Mitochondria inner membrane	ATP synthesis, ion transport, transport, embryonic development, lipid metabolism
+CA2[140]	Carbonic anhydrase II	↓	P00918	Cytoplasm, nucleus	One-carbon metabolic process, bone resorption, osteoclast differentation
*+CKB[85], [88], [90], [105], [100], [106]	Creatine kinase B-type	↑	P12277	Cytoplasm	Creatine metabolism
*MDH1[87], [141],[142]	Malate dehydrogenase, cytoplasmic	↑	P40925	Cytoplasm	Glycolysis, malate metabolism, tricarboxylic acid cycle

PROTEIN PROCESSING					
Gene	Protein name	Up/down regulation	Accession number	Cellular localization	Molecular function
*+*HSPA8* [69], [116]	Heat shock 70 kDa protein 8Heat shock cognate 71 kDa protein	↓	P11142	Cytoplasm	Chaperone, protein folding, membrane organization, post-Golgi vesicle-mediated transport
*HSPA9*	Heat shock 70 kDa protein 9 (mortalin) Stress-70 protein, mitochondrial	↑	P38646	Mitochondria	Control of cell proliferation and cellular aging; probably a chaperone
*HSPD1[69]	Heat shock 60 kDa protein 1 (chaperonin) 60 kDa heat shock protein, mitochondrial	↑↑	P10809	Mitochondria matrix	Chaperone

DEVELOPMENT					
Gene	Protein name	Up/down regulation	Accession number	Cellular localization	Molecular function
* *GAP43* [143]	Neuromodulin	↑	P17677	Cell junction, synapse, plasma membrane	Nerve growth regulation, neurogenesis, differentation, signal transduction

PROTEOLYSIS					
Gene	Protein name	Up/down regulation	Accession number	Cellular localization	Molecular function
S*CTSD[126], [125]	Cathepsin D	↑	P07339	Lysosome, melanosome	Cell death, proteolysis

GLIA CELL MARKER					
Gene	Protein name	Up/down regulation	Accession number	Cellular localization	Molecular function
S*+***GFAP*** [86], [88], [69], [13], [132], [133], [134], [119], [101]	Glial fibrillary acidic protein	↑↑↑↑↑↑	P14136	Cytolpasm, intermedier filament	Central nervous system development, structural constituent of cytoskeleton

* proteins involved in schizophrenia; +: proteins involved in depression; S: proteins involved in suicide.

* proteins involved in schizophrenia; +: proteins involved in depression; S: proteins involved in suicide. Bold-italic gene names highlighting those proteins that were found in those differently expressed protein spots that proved significant with both statistical tests. ↑ or ↓: the direction of the spot intensity change of a given spot compared to control.

Functional protein clusters of the amygdala and prefrontal cortex demonstrated both similarities and differences in the brains of suicide victims compared to controls. Of the nine proteins whose levels were altered in both the brain structures (Table 4), three (actin (ACTB), glial fibrillary acidic protein (GFAP) and cathepsin D (CTSD)) showed altered levels in opposing directions; elevated in the amygdala and lower in the cortex.

**Table 4:** Altered proteins in the prefrontal cortex and amygdala.

Gene name	Protein name	Up/down regulation in the cortex	Up/down regulation in the amygdala
**Cytoskeleton**			
ACTB*	Actin, cytoplasmic 1	↓*	↑*
***INA***	Alpha-internexin	↑↑	↑
***NEFL***	Neurofilament, light polypeptide 68 kDa	↑↑↓	↑↑↑↓
***NEFM***	Neurofilament, medium polypeptide,	↑↑↑↑↓	↑↑
***TUBA1A***	Tubulin alpha-1B chain	↑↑↓	↑↑
**Glia cell marker**			
GFAP*	Glial fibrillary acidic protein	↑#↓↓↓↓↓↓↓↓*	↑↑↑↑↑↑*
**Metabolism**			
***CKB***	Creatine kinase B-type	↑↓↓	↑
**Protein processing**			
***HSPA8***	Heat shock cognate 71 kDa protein	↑↓	↑↑↓
**Proteolysis**			
CTSD*	Cathepsin D	↓↓↓*	↑*

Proteins labelled by * were changed in both the cortex and the amygdala, but the directions of the changes were in reverse directions. Bold-italic gene name as in previous tables. ↑ or ↓: the direction of the spot intensity change of a given spot compared to control, for details see the Suppl. Materials Table 3 and 4, Suppl.

**Figure 2:**
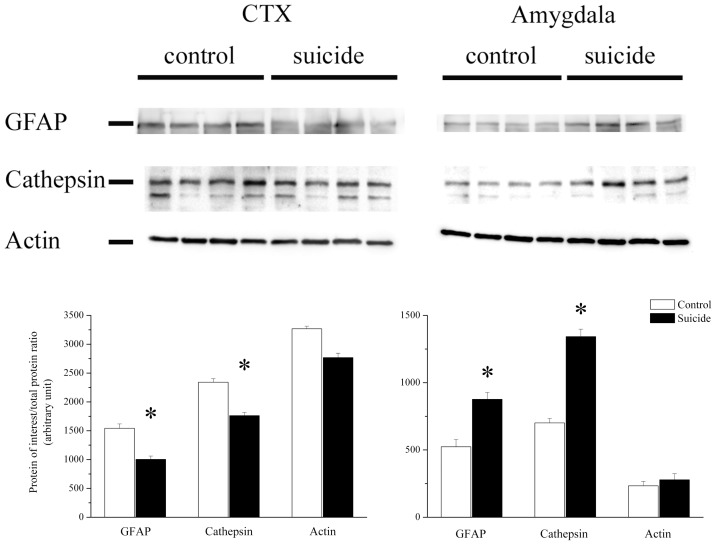
Western blot validation of GFAP, cathepsin and actin expressions in the cortex and amygdala of suicide and control subjects. The expressions of GFAP and cathepsin were significantly decreased in the suicide prefrontal cortex compared to the control samples while in the amygdala their expressions were significantly increased. In case of the actin similar but non-significant changes were found. The loading control was Ponceau, mean ± SEM.

In an attempt to validate our proteomic results, western blot analysis was carried out on the proteins that showed opposing directions of change in the two brain structures as these proteins are the most promising biomarker protein candidates, e.g. for brain imaging PET probe targets. Expressions of cathepsin (p = 0.0321) and GFAP (p = 0.0192) were significantly decreased in the suicide prefrontal cortex samples compared to the control samples, while in the amygdala, the expression of cathepsin (p = 0.0164) and GFAP (p = 0.0383) significantly increased in suicide samples (Figure 2). In case of the actin we also observed decreased level in the cortex and increased level in the amygdala of suicide samples although these changes were not significant because of high SD and low n (Figure 2).

Another set of proteins displayed parallel changes in both brain structures: creatin kinase B-type (CKB), alpha-internexin (INA), neurofilament light polypeptide (NEFL), neurofilament medium polypeptide (NEFM), tubulin alpha-1B chain (TUBA1A) and heat shock cognate 71 kDa protein (HSPA8). We did not find proteins change simultaneously in the prefrontal cortex and amygdala in functional categories: signalling, redox system and development.

Interestingly, nearly half of the altered proteins in the wider data pool had already been identified as indicative factors of suicide risk (see Table 2 and 3). In our study, we identified 35 proteins from the cortex and 16 proteins from the amygdala that had been previously linked to schizophrenia. We also identified 21 protein changes from the cortex and 9 from the amygdala that are related to depression as well as 5 proteins from the cortex and 2 proteins from the amygdala mentioned in the suicide literature (see Table 2 and 3). In this study, we identified 43 proteins from the cortex and 2 proteins from the amygdala that have never been connected to schizophrenia or depression.

## Discussion

In this study, we found changes in the expression of several proteins in the amygdala and the prefrontal cortex of suicide victims using proteomics technology. Our data reflect the widely accepted idea that suicide is the result of complex interactions of psychopathology-related molecular events [32], [33], [34], [35], [36] because several of the altered proteins have already been linked to psychiatric disorders such as schizophrenia or depression (see Table 2 and 3). Thus, our results are in agreement with the clinical observations that report coexisting psychopathological symptoms that can lead to suicide [37], [38], [39]. The proteomic changes detected in our study and the results of gene chip studies [9], [11], [40] show little overlap, which is in agreement with the fact that only a fraction of transcribed genes result in protein expression. In addition, differences in sample preparation, differences in sensitivity of protein or DNA/RNA detection and differences in the brain structures sampled may explain these differences. Similarly, the hyper-methylation of ribosomal-RNA gene promoter observed in suicide victims [14] might explain the widespread protein changes observed. Therefore, our data complement gene-chip and target-oriented mRNA studies [11], [12].

### Methodological Considerations

The applied proteomics methodology provides information on only a fraction of the proteome at one time; thus, although our results indicate certain functional processes, they do not reveal the complete functioning protein network [41]. The number of different proteins in a cell is estimated to be around 30,000, and the DIGE technology can detect only 2,000–4,000 (detecting 2,000 proteins is routine) [42], [43]. Nevertheless, the number of detected proteins is large enough to treat as a multi-spot index of change in the cellular protein network and suggests possible biomarker proteins of suicide. Additional information can be gained regarding the molecular mechanisms by linking identified proteins to known functional protein pathways of psychiatric diseases.

### Limitation of the Post Mortem Study

The proteomic analysis of *post mortem* human brain samples has some inherent limitations. The *post mortem* human brain proteome reflects the changes of protein expression in the human brain while alive, including the changes resulted from the complex psycho-pathological processes leading to suicide. However, both *pre*- and *post mortem* factors can affect tissue quality that will influence the quantitative proteomics data [44]. These factors include prolonged agonal state, metabolic state, the use of drugs, infections, hypoxia and the *post mortem* interval (PMI), that is the period from death to freezing of the brain for long-term storage [45], [46].

In our present study to decrease the effect of these factors, we used brain samples from people who had died from hanging (suicide) and from individuals who died due to acute cardiac arrest. We have to be aware that the differentially altered proteins in our study may reflect the cause of death and not solely the intended vulnerability of suicide, but this relatively homogeneous experimental sample group design is a plus in our human *post mortem* study. Moreover, the pH of each sample was measured and they had fallen in a narrow range (7.1–7.3 in lysis buffer). This could be important because the *post mortem* brain pH is informative about certain types of *ante mortem* factors [45], [47]. Furthermore, PMI was relatively short in our study (suicide group 3–6 h; control group 2–5 h) that is an advantage, although it was repeatedly demonstrated that most human brain proteins are quite stable with respect to *post mortem* factors, such as PMI [44]. Even so, we have to be aware that certain protein abundance changes are dependant on the PMI duration [44] and these proteins include e.g. GFAP and INA that also changed in this study. However, the PMI duration was short and overlapping in our study, and the spot positions in the gel and the peptide coverage of the identified protein (see Fig. S2 and Table S3, S4), as well as the opposite change of some proteins in the two brain structures, do not suggest simple protein degradation. We think, that at least some of these cytoskeleton related protein abundance changes observed in our study could be *in vivo* existing protein isoforms reflecting the pathophysiological processes of psychiatric illnesses rather than protein degradation.

However, one question is open, whether the changes in protein expression present before the suicide or the result of the trauma from the suicide. *Post mortem* brain tissue studies on suicide brains can not elucidate this question. Protein expression changes presented here can be the result of pre-suicide psychotic state, or a longer major depressive agitated state because of the long turn-over time of proteins. The hypoxia caused by hanging might not have changed the brain proteome directly because hypoxia activated proteins were not found in great number. Since we are searching for biomarkers of suicide, it would be very important to know which biomarker protein candidates are correlating with the pre-suicide psychosis however we must leave the question open.

### Extensive Protein Changes in the Brains of Suicide Victims Reflect an Altered State of Cellular Functions

Different psychiatric diseases, such as major depression [48], [49] and schizophrenia [50], may increase the risk of suicide; in turn, protein expression changes in the brains of suicide victims reflect several overlapping molecular mechanisms of different psychiatric illnesses. They may also reflect preceding psychiatric abnormalities, pre-suicide stress and/or psychopathology. Thus, we did not expect to find a pathway or protein network directly responsible for suicide; rather, we expected that molecular markers for predicting the risk for committing suicide can be uncovered. As expected, we identified several proteins already reported in the suicide and psychiatric disorder literature (see Tables 2 and 3).

Some of our results may probably indicate an altered monoaminergic neurotransmission [51] while mitochondrial enzymes, such as different ATP synthase subunits (ATP5B, ATP5C1, etc.), citrate synthase (CS), enoyl-CoA hydratase (ECHS1), and fumarate hydratase (FH) may reflect the glucose metabolism down-regulation theory of suicide [52]. On the other hand, lower amounts of peroxiredoxin 6 (PRDX6) and glutathione peroxidase (GPX1), in the brains of suicide victims support the relevance of the redox imbalance hypothesis in psychiatric patients [53]. We found changes in the expression of cytoskeleton proteins (see Tables 2 and 3), which probably reflects altered receptor trafficking and signalling [54]. Unbalanced glutamatergic and GABAergic neurotransmission are also important risk factors in developing suicide behaviour [11], [55]. Furthermore, changes in GABA_A_ receptor subunits accompanied by alterations in NMDA and AMPA receptor signalling have been found in psychopathological states related to suicide [8], [56]. Contrary to our finding in the cortices of suicide victims, decreased glutamine synthetase (GLUL) levels have been detected in schizophrenia and depression models [57], and down-regulated GLUL gene has been found among depressed suicide victims [9], [11]. This discrepancy might indicate that a suicide by hanging and its associated stress elevates excitatory events, whereas depression decreases excitatory events. Increased GLUL levels may not only indicate increased glutamate-to-glutamine conversion, but also increased glutamatergic transmission [58]. We found other proteins that indicate that elevated excitatory events may play a role in suicide; e.g. decreased cortical levels of calbindin (CALB2) suggest - as a consequence of decreased Ca^2+^ binding capacity - an elevated concentration of free Ca^2+^ that can be excitatory above a certain level [59].

Glutamine synthetase is mainly located in astrocytes, and its changes in relative level were investigated after deprivation of paradoxical sleep in rats [60]. A significant increase in GLUL level was observed e.g. in the frontoparietal cortex after paradoxical sleep deprivation that rises the issue that stress and prolonged waking could affect the physiological regulation of GLUL. In our study, it can not be excluded that the cardiac arrest in control subjects would had happened during sleep (see methods section) and the difference between those who died asleep opposed to those who died awake could influence our result. The heterogeneity of data from this regard also could increase the data dispersion. Nevertheless, we think that that the stress and the prolonged waking in case of the suicide victims could be an important issue.

In accordance with previously published changes in the GFAP of suicide victims and patients with psychotic disorders [61], we also found an increased level of GFAP in the amygdala but decreased expression in the cortex. GFAP concentration is generally believed to be an index of the number of glia cells [13]; however, astrocyte dysfunction, without a reduction in cell number, may be a factor in suicide [52]. We identified GFAP from several different gel areas (see Table 2 and 3 and Table S1, S2, S3, S4 Fig. S2), which indicates that GFAP is probably highly processed. Furthermore, a lower level of PRDX6 is known to be present in astrocytes [62]. Therefore, our data suggest that focused studies on changes in glial morphology and glial protein functions in the brains of suicide victims could be beneficial in understanding the role of glia cells in suicide.

The extensive changes detected in the proteome of suicide brain are not surprising because the ribosomal RNA level is likely decreased in the brains of suicide victims due to hyper-methylation in the RNA-promoter region [14]. Epigenetic factors, such as DNA methylation, are known to exist in different psychiatric disorders related to high suicide risk [63], [64], [65].

### Can Some Proteins be Used as Biomarker Molecules of Suicide?

Our proteomic study revealed that protein changes might be considered as a potential starting point for identifying biomarker candidates of suicide. Fifteen of the proteins we detected (carbonyl reductase [CBR1], dihydropyrimidinase-like 2 [DPYSL2], EF-hand domain family, member D2 [EFHD2], FK506-binding protein 4 [FKBP4], GFAP, GLUL, HSPA8, NEFL, NEFM, phosphoglycerate mutase 1 [PGAM1], PRDX6, SELENBP1, VIM, 14-3-3 protein eta [YWHAH] and 14-3-3 protein zeta/delta [YWHAZ]) have already been suggested as potential biomarker candidates for depression or schizophrenia [66], [67], [68], [69], [70], [71], [72], [73]. Additionally, 14-3-3 protein epsilon [YWHAE] was found to be a potential suicide susceptibility gene [74]. There were 9 protein expression changes in both the cortex and the amygdala in the brains of suicide victims compared to controls (Table 4), and four of these (GFAP, HSPA8, NEFL and NEFM) were overlapped with the previous fifteen. These 9 proteins indicate that at least some of the protein changes are global in the brains of suicide victims. Three of these proteins (ACTB, CTSD and GFAP) had opposite changes in the cortex compared to the amygdala and these opposite changes were validated by western blot analysis.

These proteins with opposite changes in the amygdala and prefrontal cortex could be particularly interesting in the scope of the functional neuroimaging studies of suicide. Greater fMRI activity of the amygdale were demonstrated on threatening stimuli in association with serotonin transporter gene promoter polymorphism [75], [76] that is known to be associated with suicidal behaviors in psychiatric patients, especially with violent suicides [77], [78]. In the prefrontal cortical regions however, lower metabolism (measured by PET) was found in association with greater suicidal ideation and greater lethality in suicide attempts in depressive patients [78], [79].

**Figure 3:**
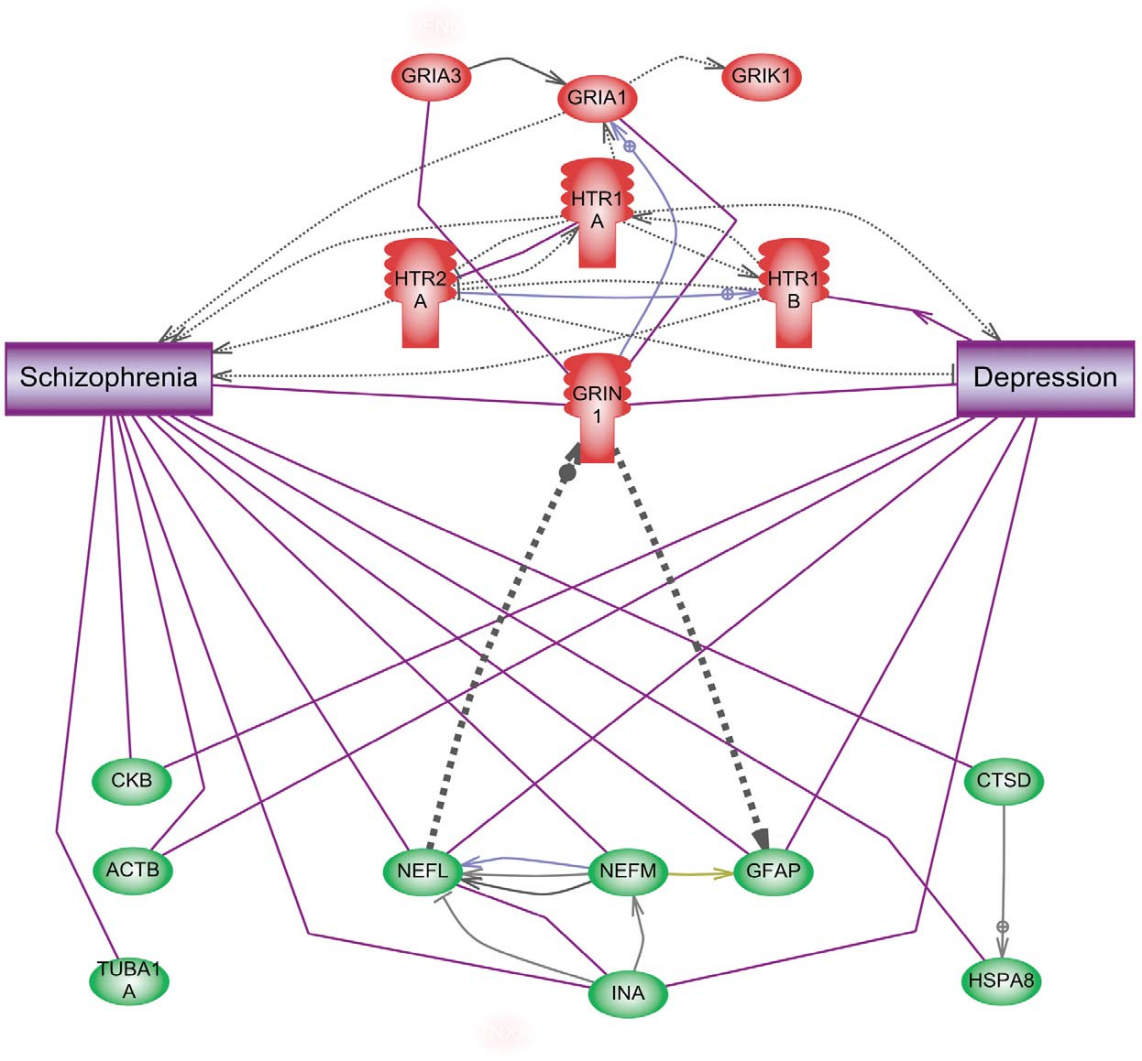
The protein network of altered cytoskeleton proteins in the brains of suicide victims (green) is connected to the receptor-interaction network of glutamate and serotonin (red) via NEFL and GFAP. Abbreviations: GRIA1– Glutamate receptor, ionotropic, AMPA1, GRIA3 - glutamate receptor, ionotrophic, AMPA 3, GRIK1– Glutamate receptor, ionotropic, kainate 1, GRIN1– Glutamate receptor, ionotropic, N-methyl-D-aspartate, HTR1A –5-Hydroxytryptamin (serotonin) receptor 1A, HTR2A (5-hydroxytryptamine (serotonin) receptor 2A, HTR1B (5-hydroxytryptamine (serotonin) receptor 1B, CKB - Creatine kinase B-type, ACTB - Actin, cytoplasmic 1, TUBA1A – Tubulin alpha-1B chain, NEFL – Neurofilament, light polypeptide 68 kDa, NEFM – Neurofilament, medium polypeptide, INA – Alpha-internexin, GFAP – Glial fibrillary acidic protein, CTSD - Cathepsin D, HSPA8 - Heat shock 70 kDa protein 8.

The protein interaction networks of the 9 proteins that changed both in the cortex and the amygdala (see Figure 3) contained a direct interaction sub-network of cytoskeletal proteins (INA, NEFL, NEFM and GFAP) which interact with binding or expression regulation. This direct interaction network of the cytoskeletal proteins is connected to the network of glutamate and serotonin receptors involved in psychotic illnesses through GRIN1 (Glutamate receptor, ionotropic, N-methyl-D-aspartate; NMDA receptor, e,g, [80]). CTSD connected to HSPA8, ACTB, CKB and TUBA1A were not directly linked to the other selected proteins. ACTB, CKB, NEFL, INA and GFAP had link to both schizophrenia and depression, while CTSD, HSPA8, NEFM and TUBA1A had link to schizophrenia.

We regard these 9 proteins as biomarker candidates of suicide risk. Furthermore, the development of quantitative brain imaging probes based on selected proteins shows promise. Prior to developing these, however, several additional studies must be performed to confirm the identity of candidate biomarkers (e.g., in other forms of suicide and in suicide trait behaviour in animals).

### Conclusion

In this study, the proteome of the prefrontal cortex changed more extensively than the amygdala of suicide victims. This result is in accordance with the fact that the prefrontal cortex is highly involved in mental disorders and suicide [81]. Because the direct interaction network of cytoskeletal proteins is changed in the brains of suicide victims, new perspectives for studying suicide-related mechanisms in receptor anchoring and ultra-structural plasticity including glia cell function have been introduced.

## Supporting Information

Figure S1 **The** ***q-*** **values were calculated from the** ***p-*** **values with the statistics software R (** www.r-project.org **; see text).** The frequency distributions of *P*-values were used to estimate the proportion of features that are unchanging; this is then used to estimate the false discovery rate. The *q*-values were graphed twice for both *p*-value range 0.0–1.0 and 0.0–0.15.
(DOC)

Figure S2 **Gel image from the prefrontal cortex, GFAP containing spots are highlighted with grey colour, the spot marked with orange is GFAP isoform containing spot. See. Table S3.**
(TIF)

Table S1 **The full list of the identified proteins by MS analysis according to spot numbers from the prefrontal cortex.** Bold gene names highlighting those proteins that were found in those differently expressed protein spots that proved significant with both statistical tests.
(DOC)

Table S2 **The full list of the identified proteins by MS analysis according to spot numbers from the amygdala.** Bold gene names highlighting those proteins that were found in those differently expressed protein spots that proved significant with both statistical tests.
(DOC)

Table S3 **The list of the identified triptic peptides of GFAP by MS analysis detected in different spots from the prefrontal cortex.**
(DOC)

Table S4 **The list of the identified triptic peptides of GFAP by MS analysis detected in different spots from the amygdala.**
(DOC)
