# Supplementary material for: Altered Functional Protein Networks in the Prefrontal Cortex and Amygdala of Victims of Suicide
Source: PLoS One. 2012 Dec 6;7(12):e50532. doi: 10.1371/journal.pone.0050532 (PMC3516509; doi:10.1371/journal.pone.0050532)
Supplement: Figure S1 — The q- values were calculated from the p- values with the statistics software R ( www.r-project.org ; see text). The frequency distributions of P-values were used to estimate the proportion of features that are unchanging; this is then used to estimate the false discovery rate. The q-values were graphed twice for both p-value range 0.0–1.0 and 0.0–0.15. (DOC) [file pone.0050532.s001.doc]

**Histogram of P−Values_PFC**

Frequency

0

50

100

150

0.0 0.2 0.4 0.6 0.8 1.0

^π^_0_ 0.158

q−value

0.04

0.12

0.16

|  |  | | | | | |
| --- | --- | --- | --- | --- | --- | --- |
|  |
|  |
|  |
|  |
|  |
|  | |  |  |  |  |  |

^ π_0_(λ)

0

0.0 0.2 0.4 0.6 0.8

expected false positives

0.0 0.2 0.4 0.6 0.8 1.0

significant tests

0

200

400

600

0.2

0.4

0.6

0.8

1.0

p−value

0

20

40

60

80100

0.08

|  |  | | | | | | |
| --- | --- | --- | --- | --- | --- | --- | --- |
|  |
|  |
|  |
|  |
|  |
|  |
|  | |  |  |  |  |  |  |

|  |  | | | | | | | |
| --- | --- | --- | --- | --- | --- | --- | --- | --- |
|  |
|  |
|  |
|  |
|  |
|  |
|  | |  |  |  |  |  |  |  |

|  |  | | | | | | |
| --- | --- | --- | --- | --- | --- | --- | --- |
|  |
|  |
|  |
|  |
|  |
|  |
|  |
|  | |  |  |  |  |  |  |

^π^_0_ 0.158

q−value

0.03

0.05

0.06

|  |  | | | | | |
| --- | --- | --- | --- | --- | --- | --- |
|  |
|  |
|  |
|  |
|  |
|  | |  |  |  |  |  |

^ π_0_(λ)

0.0 0.2 0.4 0.6 0.8

expected false positives

0.00 0.05 0.10 0.15

significant tests

0

50

100

200

0.2

0.4

0.6

0.8

1.0

p−value

0

5

10

15

0.04

|  |  | | | | |
| --- | --- | --- | --- | --- | --- |
|  |
|  |
|  |
|  |
|  | |  |  |  |  |

|  |  | | | | | | |
| --- | --- | --- | --- | --- | --- | --- | --- |
|  |
|  |
|  |
|  |
|  | |  |  |  |  |  |  |

|  |  | | | | |
| --- | --- | --- | --- | --- | --- |
|  |
|  |
|  |
|  |
|  |
|  |
|  | |  |  |  |  |

**Histogram of P−Values_AMY**

Frequency

0

20

40

60

80

100

0.0 0.2 0.4 0.6 0.8 1.0

^π^_0_ 0.738

q−value

0.20.30.40.50.6

0.7

|  |  | | | | | |
| --- | --- | --- | --- | --- | --- | --- |
|  |
|  |
|  |
|  |
|  |
|  |
|  |
|  | |  |  |  |  |  |

^ π_0_(λ)

0.0 0.2 0.4 0.6 0.8

expected false positives

100 200 300 400 500

0.0 0.2 0.4 0.6 0.8 1.0

p−value

|  |  | | | | | | |
| --- | --- | --- | --- | --- | --- | --- | --- |
|  |
|  |
|  |
|  |
|  |
|  | |  |  |  |  |  |  |

|  |  | | | | | |
| --- | --- | --- | --- | --- | --- | --- |
|  |
|  |
|  |
|  |
|  |
|  |
|  |
|  |
|  |  |  |  |  |  |  |

significant tests

0

200

400

600

0.70

0.80

0.90

1.00

0.2 0.3 0.4 0.5 0.6 0.7

0

|  |  | | | | | | | | |
| --- | --- | --- | --- | --- | --- | --- | --- | --- | --- |
|  |
|  |
|  |
|  |
|  |
|  |
|  | |  |  |  |  |  |  |  |  |

0 100 300 500 700

^π^_0_ 0.738

q−value

0.25

0.45

|  |  | | | | | |
| --- | --- | --- | --- | --- | --- | --- |
|  |
|  |
|  |
|  |
|  |
|  |
|  |
|  | |  |  |  |  |  |

^ π_0_(λ)

0.0 0.2 0.4 0.6 0.8

expected false positives

20

0.00 0.05 0.10 0.15

p−value

40

60

0.30 0.35 0.40

|  |  | | | | |
| --- | --- | --- | --- | --- | --- |
|  |
|  |
|  |
|  |
|  |
|  | |  |  |  |  |

|  |  | | | | | |
| --- | --- | --- | --- | --- | --- | --- |
|  |
|  |
|  |
|  |
|  | |  |  |  |  |  |

significant tests

0

50

100

150

0.70

0.80

0.90

1.00

0.25 0.30 0.35 0.40 0.45

0

|  |  | | | | |
| --- | --- | --- | --- | --- | --- |
|  |
|  |
|  |
|  |
|  | |  |  |  |  |

0 50 100 150
