# Supplementary figures and images for: Altered Functional Protein Networks in the Prefrontal Cortex and Amygdala of Victims of Suicide
Source: PLoS One. 2012 Dec 6;7(12):e50532. doi: 10.1371/journal.pone.0050532 (PMC3516509; doi:10.1371/journal.pone.0050532)

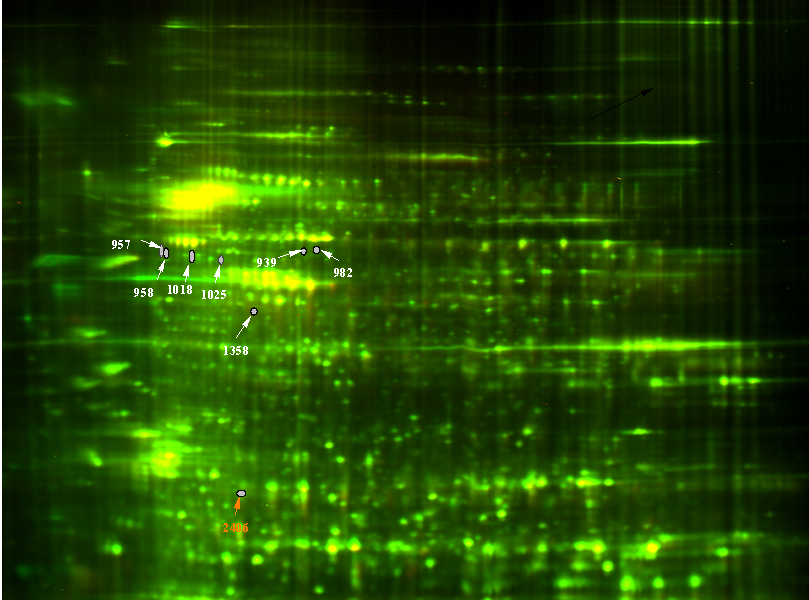

Supplement: Figure S2 — Gel image from the prefrontal cortex, GFAP containing spots are highlighted with grey colour, the spot marked with orange is GFAP isoform containing spot. See. Table S3. (TIF) [file pone.0050532.s002.tif]
