# Supplementary material for: Altered Functional Protein Networks in the Prefrontal Cortex and Amygdala of Victims of Suicide
Source: PLoS One. 2012 Dec 6;7(12):e50532. doi: 10.1371/journal.pone.0050532 (PMC3516509; doi:10.1371/journal.pone.0050532)
Supplement: Table S1 — The full list of the identified proteins by MS analysis according to spot numbers from the prefrontal cortex. Bold gene names highlighting those proteins that were found in those differently expressed protein spots that proved significant with both statistical tests. (DOC) [file pone.0050532.s003.doc]

| **Spot number** | **Gene name** | **Protein name** | **Accession number** | **p-value** | **Fold change** | **q-value** | **Number of unique peptides** | **Sequence coverage (%)** |
| --- | --- | --- | --- | --- | --- | --- | --- | --- |
|  |  |  |  |  |  |  |  |  |
| 1034 | **PGK1** | Phosphoglycerate kinase 1 | P00558 | 0.01644 | 2.2019 | **0.059415** | 24 | 64 |
|  | **IDH2** | Isocitrate dehydrogenase [NADP], mitochondrial | P48735 |  |  |  | 14 | 34 |
|  | **UQCRC2** | Cytochrome b-c1 complex subunit 2, mitochondrial | P22695 |  |  |  | 7 | 21 |
| 1031 | **PGK1** | Phosphoglycerate kinase 1 | P00558 | 0.007517 | 2.1428 | **0.055402** | 18 | 51 |
|  | **TUBA1A** | Tubulin alpha-1A chain | Q71U36 |  |  |  | 10 | 29 |
|  | **CS** | Citrate synthase, mitochondrial | O75390 |  |  |  | 8 | 19 |
| 1032 | **PGK1** | Phosphoglycerate kinase 1 | P00558 | 0.01253 | 2.0377 | **0.059415** | 19 | 51 |
|  | **UQCRC2** | Cytochrome b-c1 complex subunit 2, mitochondrial | P22695 |  |  |  | 13 | 34 |
|  | **CS** | Citrate synthase, mitochondrial | O75390 |  |  |  | 7 | 16 |
|  | **IDH2** | Isocitrate dehydrogenase [NADP], mitochondrial | P48735 |  |  |  | 4 | 9 |
| 1081 | SEPT5 | Septin-5 | Q99648 | 0.0418 | 2.032 | 0.062862 | 10 | 22 |
|  | SEPT2 | Septin-2 | Q15019 |  |  |  | 5 | 18 |
|  | SEPT3 | Neuronal-specific septin-3 | Q9UH03 |  |  |  | 5 | 15 |
|  | PDHA1 | Pyruvate dehydrogenase E1 component subunit alpha, somatic form, mitochondrial | P08559 |  |  |  | 4 | 11 |
| 1023 | **FH** | Fumarate hydratase, mitochondrion | P07954 | 0.006892 | 2.0048 | **0.055402** | 10 | 26 |
|  | **UQCRC2** | Cytochrome b-c1 complex subunit 2, mitochondrial | P22695 |  |  |  | 10 | 26 |
|  | **SYN1** | Synapsin-1 | P17600 |  |  |  | 4 | 6 |
| 1090 | **PDHA1** | Pyruvate dehydrogenase E1 component subunit alpha, somatic form, mitochondrial | P08559 | 0.01137 | 1.9771 | **0.059415** | 7 | 13 |
|  | **SEPT3** | Neuronal-specific septin-3 | Q9UH03 |  |  |  | 5 | 16 |
|  | **GOT1** | Aspartate aminotransferase, cytoplasmic | P17174 |  |  |  | 4 | 12 |
|  | **GLUL** | Glutamine synthetase | P15104 |  |  |  | 4 | 16 |
|  | **C5orf33** | UPF0465 protein C5orf33 | Q4G0N4 |  |  |  | 4 | 18 |
|  | **SEPT5** | Septin 5 | Q99719 |  |  |  | 3 | 9 |
| 982 | **GFAP** | Glial fibrillary acidic protein isoform 1 | P14136 | 0.02106 | 1.9423 | **0.055402** | 29 | 56 |
| 939 | **GFAP** | Glial fibrillary acidic protein isoform 1 | P14136 | 0.02032 | -1.859 | **0.055402** | 39 | 61 |
| 1102 | UQCRC2 | Cytochrome b-c1 complex subunit 2, mitochondrial | P22695 | 0.04252 | 1.7271 | 0.062862 | 13 | 33 |
|  | PGK1 | Phosphoglycerate kinase 1 | P00558 |  |  |  | 9 | 28 |
|  | CS | Citrate synthase, mitochondrial | O75390 |  |  |  | 4 | 9 |
| 2523 | PSMB4 | Proteasome subunit beta type-4 | P28070 | 0.02451 | -1.7657 | 0.062862 | 10 | 36 |
| 582 | **DPYSL2** | Dihydropyrimidinase-like 2 | Q16555 | 0.004775 | 1.7521 | **0.055402** | 25 | 55 |
|  | **ATP6V1B2** | V-type proton ATPase subunit B, brain isoform | P21281 |  |  |  | 9 | 24 |
|  | **PDIA3** | Protein disulfide-isomerase A3 | P30101 |  |  |  | 8 | 18 |
|  | **NEFM** | Neurofilament medium polypeptide | P07197 |  |  |  | 6 | 7 |
|  | **TUBA1C** | Tubulin alpha-1C chain | Q9BQE3 |  |  |  | 3 | 8 |
| 1795 | VDAC1 | Voltage-dependent anion channel 1 | P21796 | 0.02902 | 1.7516 | 0.062862 | 14 | 56 |
|  | CBR1 | Carbonyl reductase [NADPH] 1 | P16152 |  |  |  | 11 | 45 |
|  | HADH | Hydroxyacyl-coenzyme A dehydrogenase, mitochondrial | Q16836 |  |  |  | 4 | 10 |
| 2531 | **GRB2** | Growth factor receptor-bound protein 2 | P62993 | 0.0146 | -1.7516 | **0.059415** | 9 | 39 |
|  | **SERPINB3** | Serpin B3 | P29508 |  |  |  | 3 | 9 |
| 2413 | **ECHS1** | Enoyl-CoA hydratase | P30084 | 0.002836 | -1.7448 | **0.055402** | 16 | 38 |
|  | **HSPB1** | Heat shock protein beta-1 | P04792 |  |  |  | 8 | 36 |
|  | **PGLS** | 6-Phosphogluconolactonase | O95336 |  |  |  | 7 | 34 |
| 580 | **INA** | alpha-Internexin | Q16352 | 0.01967 | 1.7056 | **0.059415** | 18 | 35 |
|  | **FKBP4** | FK506-binding protein 4 | Q02790 |  |  |  | 5 | 15 |
| 812 | **DPYSL2** | Dihydropyrimidinase-like 2 | Q16555 | 0.005068 | 1.6707 | **0.055402** | 5 | 11 |
|  | **PDIA3** | Protein disulfide-isomerase A3 | P30101 |  |  |  | 5 | 11 |
| 773 | SELENBP1 | Selenium binding protein 1 | Q13228 | 0.02958 | 1.6658 | 0.062862 | 16 | 38 |
|  | NEFM | Neurofilament medium polypeptide | P07197 |  |  |  | 13 | 17 |
|  | PDHX | Pyruvate dehydrogenase protein X component, mitochondrial | O00330 |  |  |  | 5 | 11 |
| 106 | NEFL | Neurofilament, light polypeptide 68kDa | P07196 | 0.02979 | 1.6409 | 0.062862 | 33 | 40 |
|  | TUBA1B | Tubulin alpha-1B chain | P68363 |  |  |  | 6 | 19 |
| 1149 | **PURA** | Transcriptional activator protein Pur-alpha | Q00577 | 0.01716 | 1.6331 | **0.059415** | 7 | 23 |
|  | **GLUL** | Glutamine synthetase | P15104 |  |  |  | 7 | 25 |
|  | **PDHA1** | Pyruvate dehydrogenase E1 component subunit alpha, somatic form, mitochondrial | P08559 |  |  |  | 4 | 9 |
|  | **MAPK3** | Mitogen-activated protein kinase 3 | P27361 |  |  |  | 3 | 11 |
|  | **PKM2** | Pyruvate kinase isozymes M1/M2 | P14618 |  |  |  | 4 | 9 |
| 2422 | **UCHL1** | Ubiquitin carboxyl-terminal hydrolase isozyme L1 | P09936 | 0.007654 | 1.6232 | **0.055402** | 18 | 78 |
|  | **GSTM3** | Glutathione S-transferase Mu 3 | P21266 |  |  |  | 8 | 46 |
|  | **APOA1BP** | Apolipoprotein A-I-binding protein | Q8NCW5 |  |  |  | 5 | 30 |
| 1157 | GOT1 | Aspartate aminotransferase, cytoplasmic | P17174 | 0.026 | 1.6199 | 0.062862 | 14 | 45 |
| 1500 | YWHAZ | 14-3-3 protein zeta/delta | P63104 | 0.03289 | 1.6159 | 0.062862 | 13 | 49 |
|  | YWHAB | 14-3-3 protein beta/alpha | P31946 |  |  |  | 7 (12) | 18(31) |
|  | YWHAH | 14-3-3 protein eta | Q04917 |  |  |  | 7 (10) | 27(33) |
|  | YWHAE | 14-3-3 protein epsilon | P62258 |  |  |  | 2(4) | 9(12) |
| 1732 | **SIRT2** | NAD-dependent deacetylase sirtuin-2 | Q8IXJ6 | 0.01293 | 1.6097 | **0.059415** | 9 | 23 |
|  | **IDH3A** | Isocitrate dehydrogenase [NAD] subunit alpha, mitochondrial | P50213 |  |  |  | 7 | 16 |
|  | **TALDO1** | Transaldolase | P37837 |  |  |  | 3 | 9 |
|  | **VTA1** | Vacuolar protein sorting-associated protein VTA1 homolog | Q9NP79 |  |  |  | 3 | 11 |
| 2387 | **PGLS** | 6-Phosphogluconolactonase | O95336 | 0.00867 | -1.6093 | **0.055402** | 11 | 55 |
|  | **ECHS1** | Enoyl-CoA hydratase, mitochondrial | P30084 |  |  |  | 6 | 24 |
| 2436 | **HNRPDL** | Heterogeneous nuclear ribonucleoprotein D-like | O14979 | 0.01121 | 1.5589 | **0.059415** | 4 | 9 |
|  | **APOL2** | Apolipoprotein L2 | Q9BQE5 |  |  |  | 3 | 9 |
|  | **CRKL** | Crk-like protein | P46109 |  |  |  | 3 | 13 |
| 2082 | **EFHD2** | EF-hand domain-containing protein D2 | Q96C19 | 0.004931 | -1.5475 | **0.055402** | 17 | 56 |
|  | **ACTB** | Actin, cytoplasmic 1 | P60709 |  |  |  | 3 | 6 |
|  | **CTSD** | Cathepsin D | P07339 |  |  |  | 4 | 9 |
| 2152 | **PGAM1** | Phosphoglycerate mutase 1 | P18669 | 0.01781 | -1.5292 | **0.059415** | 7 | 33 |
|  | **PRDX6** | Peroxiredoxin 6 | P30041 |  |  |  | 3 | 14 |
| 2117 | **TPD52** | Tumor protein D52 | P55327 | 0.01458 | -1.5284 | **0.059415** | 7 | 36 |
|  | **CTSD** | Cathepsin D | P07339 |  |  |  | 8 | 18 |
|  | **CKB** | Creatine kinase B-type | P12277 |  |  |  | 5 | 18 |
|  | **NEFL** | Neurofilament light polypeptide | P07196 |  |  |  | 6 | 12 |
|  | **PHB** | Prohibitin | P35232 |  |  |  | 3 | 10 |
| 2454 | CAPNS1 | Calpain small subunit 1 | P04632 | 0.01912 | -1.5253 | 0.060317 | 7 | 20 |
|  | CKB | Creatine kinase | P12277 |  |  |  | 3 | 9 |
|  | TUBA1A | Tubulin alpha-1A chain | Q71U36 |  |  |  | 3 | 7 |
|  | GFAP | Glial fibrillary acidic protein isoform 1 | P14136 |  |  |  | 3 | 7 |
| 761 | **NEFM** | Neurofilament medium polypeptide | Q4QRK6 | 0.00623 | 1.5124 | **0.055402** | 16 | 21 |
|  | **ATP6V1B2** | V-type proton ATPase subunit B, brain isoform | P21281 |  |  |  | 11 | 27 |
|  | **DPYSL2** | Dihydropyrimidinase-like 2 | Q16555 |  |  |  | 10 | 23 |
|  | **SELENBP1** | Selenium-binding protein 1 | Q13228 |  |  |  | 8 | 18 |
|  | **CAP2** | Adenylyl cyclase-associated protein 2 | P40123 |  |  |  | 4 | 9 |
| 2406 | **APOA1** | Apolipoprotein A-I | P02647 | 0.007494 | 1.5002 | **0.055402** | 15 | 51 |
|  | **UCHL1** | Ubiquitin carboxyl-terminal hydrolase isozyme L1 | P09936 |  |  |  | 8 | 41 |
|  | **GFAP** | Glial fibrillary acidic protein, isoform CRA_a | P14136 |  |  |  | 5 | 19 |
|  | **HSPA8** | Heat shock cognate 71 kDa protein | P11142 |  |  |  | 5 | 15 |
|  | **GSTM3** | Glutathione S-transferase Mu 3 (brain) | P21266 |  |  |  | 4 | 19 |
| 1786 | **VDAC1** | Voltage-dependent anion channel 1 | P21796 | 0.002986 | 1.4975 | **0.055402** | 9 | 41 |
|  | **CBR1** | Carbonyl reductase [NADPH] 1 | P16152 |  |  |  | 7 | 26 |
|  | **VDAC2** | Voltage-dependent anion channel 2 | P45880 |  |  |  | 5 | 18 |
| 1018 | GFAP | Glial fibrillary acidic protein isoform 1 | P14136 | 0.02779 | -1.457 | 0.062862 | 31 | 57 |
|  | ATP5B | ATP synthase subunit beta, mitochondrial | P06576 |  |  |  | 5 | 9 |
|  | TUBB4 | Tubulin beta-4 chain | P04350 |  |  |  | 4 | 9 |
|  | TUBA1C | Tubulin alpha-1C chain | Q9BQE3 |  |  |  | 3 | 8 |
|  | ATP6V1B2 | V-type proton ATPase subunit B, brain isoform | P21281 |  |  |  | 3 | 9 |
| 2218 | CKB | Creatine kinase B-type | P12277 | 0.04151 | -1.4524 | 0.062862 | 8 | 26 |
|  | PGAM1 | Phosphoglycerate mutase 1 | P18669 |  |  |  | 7 | 33 |
|  | VDAC1 | Voltage-dependent anion channel 1 | P21796 |  |  |  | 3 | 13 |
| 2169 | GFAP | Glial fibrillary acidic protein | P14136 | 0.04722 | -1.4273 | 0.062862 | 15 | 36 |
| 2234 | **YWHAG** | 14-3-3 protein gamma | P61981 | 0.01381 | -1.42 | **0.059415** | 10 | 25 |
|  | **CALB2** | Calretinin | P22676 |  |  |  | 3 | 11 |
| 734 | ATP6V1B2 | V-type proton ATPase subunit B, brain isoform | P21281 | 0.04406 | 1.4161 | 0.062862 | 22 | 41 |
|  | NEFM | Neurofilament medium polypeptide | Q4QRK6 |  |  |  | 18 | 23 |
|  | TUBA1A | Tubulin alpha-1A chain | Q71U36 |  |  |  | 10 | 29 |
| 2802 | ATP5H | ATP synthase subunit d, mitochondrial | O75947 | 0.02174 | -1.4155 | 0.062862 | 10 | 60 |
| 1502 | CAPZA2 | F-actin-capping protein subunit alpha-2 | P47755 | 0.04211 | 1.401 | 0.062862 | 11 | 59 |
|  | BRCC3 | Lys-63-specific deubiquitinase BRCC36 | P46736 |  |  |  | 3 | 12 |
| 1403 | ALDOC | Fructose bisphosphate aldolase C | P09972 | 0.02965 | 1.3978 | 0.062862 | 13 | 33 |
| 2089 | EFHD2 | EF-hand domain family, member D2 | Q96C19 | 0.02135 | -1.3736 | 0.062862 | 15 | 51 |
|  | IMPA1 | Inositol monophosphatase | P29218 |  |  |  | 5 | 18 |
|  | CTSD | Cathepsin D | P07339 |  |  |  | 6 | 13 |
|  | TPD52L2 | Tumor protein D54 | O43399 |  |  |  | 3 | 26 |
| 431 | **NDUFS1** | NADH-ubiquinone oxidoreductase 75 kDa subunit, mitochondrial | P28331 | 0.008781 | -1.355 | **0.055402** | 25 | 40 |
| 2641 | PARK7 | Protein DJ-1 | Q99497 | 0.03638 | -1.3416 | 0.062862 | 11 | 44 |
|  | NDUFV2 | NADH dehydrogenase FeS protein | Q6IPW4 |  |  |  | 9 | 28 |
|  | GPX1 | Glutathione peroxidase | P07203 |  |  |  | 6 | 43 |
|  | GUK1 | Guanylate kinase | Q16774 |  |  |  | 7 | 33 |
|  | HSPA8 | Heat shock cognate 71 kDa protein | P11142 |  |  |  | 4 | 10 |
|  | ABHD14B | Abhydrolase domain-containing protein 14B | Q96IU4 |  |  |  | 4 | 24 |
| 717 | **PKM2** | Pyruvate kinase isozymes M1/M2 | P14618 | 0.01732 | 1.3348 | **0.059415** | 3 | 7 |
| 1938 | **VDAC1** | Voltage-dependent anion channel 1 | P21796 | 0.0126 | -1.3301 | **0.059415** | 16 | 63 |
|  | **CBR1** | Carbonyl reductase 1 | P16152 |  |  |  | 8 | 35 |
|  | **ATP5C1** | ATP synthase subunit gamma, mitochondrial | P36542 |  |  |  | 3 | 11 |
| 1793 | GLOD4 | Glyoxalase domain-containing protein 4 | Q9HC38 | 0.0486 | 1.3233 | 0.062862 | 16 | 41 |
|  | NEFL | Neurofilament light polypeptide | P07196 |  |  |  | 5 | 10 |
|  | TUBA1C | Tubulin alpha-1C chain | Q9BQE3 |  |  |  | 3 | 10 |
|  | VDAC2 | Voltage-dependent anion channel 2 | P45880 |  |  |  | 3 | 15 |
|  | ATP6V1D | V-type proton ATPase subunit D | Q9Y5K8 |  |  |  | 3 | 14 |
