# Supplementary material for: Altered Functional Protein Networks in the Prefrontal Cortex and Amygdala of Victims of Suicide
Source: PLoS One. 2012 Dec 6;7(12):e50532. doi: 10.1371/journal.pone.0050532 (PMC3516509; doi:10.1371/journal.pone.0050532)
Supplement: Table S2 — The full list of the identified proteins by MS analysis according to spot numbers from the amygdala. Bold gene names highlighting those proteins that were found in those differently expressed protein spots that proved significant with both statistical tests. (DOC) [file pone.0050532.s004.doc]

| **Spot number** | **Gene name** | **Protein name** | **Accession number** | **p-value** | **Fold change** | **q-value** | **Number of unique peptides** | **Sequence coverage (%)** |
| --- | --- | --- | --- | --- | --- | --- | --- | --- |
| 320 | HSPD1 | 60 kDa heat shock protein, mitochondrial | P10809 | 0.017 | 2.42 | 0.452336 | 4 | 5% |
| 1053 | **GFAP** | Glial fibrillary acidic protein | P14136 | 0.008167 | 2.3614 | **0.217159** | 22 | 44% |
|  | **NEFL** | Neurofilament, light polypeptide 68kDa | P07196 |  |  |  | 5 | 10% |
| 192 | **HSPA8** | Heat shock cognate 71 kDa protein | P11142 | 0.00403 | -2.1632 | **0.21797** | 6 | 8% |
| 322 | **INA** | Alpha-internexin (66kDa neurofilament protein) | Q16352 | 0.003383 | 1.9225 | **0.217159** | 9 | 19% |
|  | **NEFM** | NEFM protein | Q4QRK6 |  |  |  | 6 | 6% |
| 895 | GFAP | Glial fibrillary acidic protein | P14136 | 0.045 | 1.7532 | 0.452336 | 12 | 22% |
|  | NEFL | Neurofilament, light polypeptide 68kDa | P07196 |  |  |  | 3 | 5% |
| 366 | **VIM** | Vimentin | P08670 | 0.004456 | 1.7269 | **0.21797** | 4 | 7% |
|  | **TUBA1A** | Tubulin alpha-1A chain | Q71U36 |  |  |  | 6 | 12% |
| 955 | **GAP43** | Neuromodulin | P17677 | 0.01304 | 1.6767 | **0.393907** | 6 | 39% |
| 877 | NEFL | Neurofilament, light polypeptide 68kDa | P07196 | 0.045 | -1.6432 | 0.452336 | 4 | 7% |
| 657 | GFAP | Glial fibrillary acidic protein | P14136 | 0.0403 | 1.6200 | 0.452336 | 3 | 6% |
| 1092 | **GFAP** | Glial fibrillary acidic protein | P14136 | 0.0048 | 1.55 | **0.21797** | 23 | 40% |
|  | **CKB** | Creatine kinase B-type | P12277 |  |  |  | 4 | 15% |
|  | **NEFL** | Neurofilament, light polypeptide 68kDa | P07196 |  |  |  | 4 | 7% |
|  | **ACTB** | Actin, cytoplasmic 1 | P60709 |  |  |  | 5 | 15% |
| 1645 | EFHD2 | EF-hand domain family, member D2 | Q96C19 | 0.034 | 1.5355 | 0.452336 | 6 | 23% |
|  | CTSD | Cathepsin D | P07339 |  |  |  | 7 | 15% |
|  | PHB | Prohibitin | P35232 |  |  |  | 4 | 15% |
|  | TUBB3 | Tubulin beta-3 chain | Q13509 |  |  |  | 4 | 8% |
|  | ANXA5 | Annexin A5 | P08758 |  |  |  | 3 | 9% |
| 502 | **ATP5A1** | ATP synthase subunit alpha, mitochondrial (precursor) | P25705 | 0.003313 | -1.5128 | **0.217159** | 17 | 38% |
| 417 | **GFAP** | Glial fibrillary acidic protein | P14136 | 0.004669 | 1.4088 | **0.21797** | 19 | 33% |
|  | **HSPA9** | Stress-70 protein, mitochondrial | P38646 |  |  |  | 5 | 4% |
| 1654 | CA2 | Carbonic anhydrase II | P00918 | 0.036 | -1.39 | 0.452336 | 4 | 11% |
| 283 | **TUBA1A** | Tubulin alpha-1A chain | Q71U36 | 0.0118 | 1.3798 | **0.393907** | 3 | 7% |
| 1096 | GFAP | Glial fibrillary acidic protein | P14136 | 0.03611 | 1.355 | 0.452336 | 26 | 40% |
|  | NEFL | Neurofilament, light polypeptide 68kDa | P07196 |  |  |  | 8 | 14% |
|  | MDH1 | Malate dehydrogenase, cytoplasmic | P40925 |  |  |  | 4 | 13% |
