# Supplementary material for: Altered Functional Protein Networks in the Prefrontal Cortex and Amygdala of Victims of Suicide
Source: PLoS One. 2012 Dec 6;7(12):e50532. doi: 10.1371/journal.pone.0050532 (PMC3516509; doi:10.1371/journal.pone.0050532)
Supplement: Table S3 — The list of the identified triptic peptides of GFAP by MS analysis detected in different spots from the prefrontal cortex. (DOC) [file pone.0050532.s005.doc]

**Prefrontal cortex**

**GFAP**

glial fibrillary acidic protein [Homo sapiens]Nominal mass (Mr): 49907; Calculated pI value: 5.42

**Spot number 939**

**T-test value 0.02032**

**Up/down regulation -1.859**

**Spot number 957**

**T-test value 0.00868**

**Up/down regulation -1.2772**

**Spot number 958**

**T-test value 0.02533**

**Up/down regulation -1.2061**

**Spot number 982**

**T-test value 0.02106**

**Up/down regulation -1.9423**

**Spot number 1018**

**T-test value 0.02779**

**Up/down regulation -1.457**

**Spot number 1025**

**T-test value 0.00875**

**Up/down regulation -1.2771**

**Spot number 1358**

**T-test value 0.00821**

**Up/down regulation -1.1985**

**Spot number 1449**

**T-test value 0.03153**

**Up/down regulation -1.5811**

| 1 | MERRRITSAA | RRSYVSSGEM | MVGGLAPGRR | LGPGTRLSLA | RMPPPLPTRV |
| --- | --- | --- | --- | --- | --- |
| 51 | DFSLAGALNA | GFKETRASER | AEMMELNDRF | ASYIEKVRFL | EQQNKALAAE |
| 101 | LNQLRAKEPT | KLADVYQAEL | RELRLRLDQL | TANSARLEVE | DNLAQDLAT |
| 151 | VRQKLQDETN | LRLEAENNLA | AYRQEADEAT | LARLDLERKI | ESLEEEIRFL |
| 201 | RKIHEEEVRE | LQEQLARQQV | HVELDVAKPD | LTAALKEIRT | YEAMASSNM |
| 251 | HEAEEWYRSK | FADLTDAAAR | NAELLRQAKH | EANDYRRQLQ | LTCDLESLR |
| 301 | GTNESLERQM | REQEERHVRE | AASYQEALAR | LEEEGQSLKD | MARHLQEYQ |
| 351 | DLLNVKLALD | IEIATYRKLL | EGEENRITIP | VQTFSNLQIR | ETSLDTKSVS |
| 401 | EGHLKRNIVV | KTVEMRDGEV | IKESKQEHKD | VM |  |

|  |  | **939** | **957** | **958** | **982** | **1018** | **1025** | **1358** | **1449** |
| --- | --- | --- | --- | --- | --- | --- | --- | --- | --- |
| **12 - 29** | **R.RSYVSSGEMMVGGLAPGR.R** | **X** |  |  | **X** |  |  | **X** |  |
| **13 - 29** | **R.SYVSSGEMMVGGLAPGR.R** | **X** |  |  | **X** |  |  |  |  |
| **13 - 29** | **R.SYVSSGEMMVGGLAPGR.R** Oxidation (M) | **X** |  |  | **X** |  |  |  |  |
| **13 - 30** | **R.SYVSSGEMMVGGLAPGRR.L** | **X** |  |  | **X** |  |  |  |  |
| **42 - 63** | **R.MPPPLPTRVDFSLAGALNAGFK.E** Oxidation (M) | **X** |  |  |  |  |  |  |  |
| **42 - 66** | **R.MPPPLPTRVDFSLAGALNAGFKETR.A** | **X** |  |  |  |  |  |  |  |
| **50 - 63** | **R.VDFSLAGALNAGFK.E** | **X** |  |  | **X** | **X** | **X** | **X** |  |
| **50 - 66** | **R.VDFSLAGALNAGFKETR.A** | **X** |  |  |  |  |  |  |  |
| **71 - 79** | **R.AEMMELNDR.F** | **X** |  |  |  | **X** | **X** |  | **X** |
| **71 - 79** | **R.AEMMELNDR.F** Oxidation (M) | **X** |  | **X** | **X** | **X** | **X** |  | **X** |
| **71 - 79** | **R.AEMMELNDR.F** 2 Oxidation (M) | **X** |  |  | **X** | **X** |  |  | **X** |
| **96 - 105** | **K.ALAAELNQLR.A** | **X** | **X** | **X** |  | **X** | **X** | **X** | **X** |
| **112 - 121** | **K.LADVYQAELR.E** |  | **X** | **X** |  | **X** | **X** | **X** | **X** |
| **125 - 136** | **R.LRLDQLTANSAR.L** | **X** |  | **X** |  | **X** | **X** | **X** | **X** |
| **127 - 136** | **R.LDQLTANSAR.L** | **X** |  |  |  | **X** | **X** |  | **X** |
| **137 - 152** | **R.LEVERDNLAQDLATVR.Q** | **X** |  | **X** |  | **X** | **X** | **X** | **X** |
| **142 - 152** | **R.DNLAQDLATVR.Q** | **X** |  | **X** | **X** | **X** | **X** | **X** | **X** |
| **153 - 162** | **R.QKLQDETNLR.L** | **X** |  |  | **X** | **X** | **X** |  | **X** |
| **153 - 162** | **R.QKLQDETNLR.L** Gln->pyro-Glu (N-term Q) |  |  |  |  |  |  |  | **X** |
| **155 - 162** | **K.LQDETNLR.L** | **X** |  |  |  | **X** |  |  |  |
| **163 - 183** | **R.LEAENNLAAYRQEADEATLAR.L** | **X** |  | **X** | **X** | **X** | **X** | **X** | **X** |
| **174 - 183** | **R.QEADEATLAR.L** | **X** |  | **X** | **X** | **X** | **X** | **X** | **X** |
| **174 - 183** | **R.QEADEATLAR.L** Gln->pyro-Glu (N-term Q) |  |  |  |  |  |  |  | **X** |
| **189 - 198** | **R.KIESLEEEIR.F** | **X** |  | **X** |  | **X** | **X** | **X** | **X** |
| **190 - 198** | **K.IESLEEEIR.F** | **X** |  |  | **X** |  | **X** |  |  |
| **202 - 209** | **R.KIHEEEVR.E** | **X** |  |  |  |  |  |  |  |
| **203 - 217** | **R.KIHEEEVRELQEQLAR.Q** |  |  |  |  | **X** |  |  |  |
| **218 - 236** | **R.QQVHVELDVAKPDLTAALK.E** | **X** |  |  | **X** | **X** |  |  | **X** |
| **218 - 236** | **R.QQVHVELDVAKPDLTAALK.E** Gln->pyro-Glu (N-term Q) | **X** |  |  | **X** |  | **X** |  |  |
| **240 - 258** | **R.TQYEAMASSNMHEAEEWYR.S** | **X** |  |  | **X** |  |  |  |  |
| **240 - 258** | **R.TQYEAMASSNMHEAEEWYR.S** Oxidation (M) | **X** |  |  |  |  |  |  |  |
| **259 - 270** | **R.SKFADLTDAAAR.N** | **X** |  |  | **X** | **X** | **X** |  | **X** |
| **261 - 270** | **K.FADLTDAAAR.N** |  | **X** | **X** |  | **X** | **X** | **X** | **X** |
| **288 - 300** | **R.QLQSLTCDLESLR.G** | **X** |  | **X** |  | **X** | **X** | **X** | **X** |
| **288 - 300** | **R.QLQSLTCDLESLR.G** Gln->pyro-Glu (N-term Q) | **X** |  |  | **X** | **X** |  | **X** |  |
| **288 - 308** | **R.QLQSLTCDLESLRGTNESLER.Q** |  |  |  |  | **X** |  |  |  |
| **320 - 330** | **R.EAASYQEALAR.L** | **X** | **X** | **X** | **X** | **X** | **X** | **X** | **X** |
| **331 - 344** | **R.LEEEGQSLKDEMAR.H** | **X** |  | **X** | **X** | **X** | **X** |  | **X** |
| **331 - 344** | **R.LEEEGQSLKDEMAR.H** Oxidation (M) | **X** |  |  |  | **X** |  |  | **X** |
| **345 - 356** | **R.HLQEYQDLLNVK.L** | **X** | **X** | **X** | **X** | **X** | **X** | **X** | **X** |
| **357 - 367** | **K.LALDIEIATYR.K** | **X** |  | **X** | **X** | **X** | **X** | **X** | **X** |
| **357 - 368** | **K.LALDIEIATYRK.L** | **X** |  |  |  | **X** |  |  |  |
| **368 - 376** | **R.KLLEGEENR.I** | **X** |  | **X** | **X** | **X** |  |  | **X** |
| **368 - 390** | **R.KLLEGEENRITIPVQTFSNLQIR** | **X** |  |  | **X** |  |  |  |  |
| **369 - 376** | **K.LLEGEENR.I** | **X** |  |  |  | **X** | **X** | **X** | **X** |
| **369 - 390** | **K.LLEGEENRITIPVQTFSNLQIR.E** | **X** |  |  |  |  |  |  |  |
| **377 - 390** | **R.ITIPVQTFSNLQIR.E** | **X** | **X** | **X** | **X** | **X** | **X** |  | **X** |

**Spot number 2406**

**T-test value 0.00749**

**Up/down regulation 1.5002**

**glial fibrillary acidic protein, isoform CRA_a** [Homo sapiens]

Nominal mass (Mr): 36505; Calculated pI value: 5.55;

| 1 | MATHPNTAPK | LSPPEQCAAP | HCPRRRGKAR | LRNGEKEPGA | PVALYSSPLL |
| --- | --- | --- | --- | --- | --- |
| 51 | PLQEADEATL | ARLDLERKIE | SLEEEIRFLR | KIHEEEVREL | QEQLARQQVH |
| 101 | VELDVAKPDL | TAALKEIRTQ | YEAMASSNMH | EAEEWYRSKF | DLTDAAARN |
| 151 | AELLRQAKHE | ANDYRRQLQS | LTCDLESLRG | TNESLERQMR | EQEERHVREA |
| 201 | ASYQEALARL | EEEGQSLKDE | MARHLQEYQD | LLNVKLALDI | EIATYRKLLE |
| 251 | GEENRITIPV | QTFSNLQIRG | QYSRASWEGH | WSPAPSSRAC | RLLQTGTEDQ |
| 301 | GKGIQLSLGA | FVTLQRS |  |  |  |

|  |  |  |  |  |  |  |  |  |  | **2406** |
| --- | --- | --- | --- | --- | --- | --- | --- | --- | --- | --- |
| **167 - 179** | **R.QLQSLTCDLESLR.G** |  |  |  |  |  |  |  |  | **X** |
| **199 - 209** | **R.EAASYQEALAR.L** |  |  |  |  |  |  |  |  | **X** |
| **224 - 235** | **R.HLQEYQDLLNVK.L** |  |  |  |  |  |  |  |  | **X** |
| **236 - 246** | **K.LALDIEIATYR.K** |  |  |  |  |  |  |  |  | **X** |
| **256 - 269** | **R.ITIPVQTFSNLQIR.*G*** |  |  |  |  |  |  |  |  | **X** |
