# Supplementary material for: Altered Functional Protein Networks in the Prefrontal Cortex and Amygdala of Victims of Suicide
Source: PLoS One. 2012 Dec 6;7(12):e50532. doi: 10.1371/journal.pone.0050532 (PMC3516509; doi:10.1371/journal.pone.0050532)
Supplement: Table S4 — The list of the identified triptic peptides of GFAP by MS analysis detected in different spots from the amygdala. (DOC) [file pone.0050532.s006.doc]

**Prefrontal cortex**

**GFAP**

glial fibrillary acidic protein [Homo sapiens]Nominal mass (Mr): 49907; Calculated pI value: 5.42

**Spot number 368**

**T-test value 0.03707**

**Up/down regulation 1.9089**

**Spot number 417**

**T-test value 0.004669**

**Up/down regulation 1.4088**

**Spot number 657**

**T-test value 0.0403**

**Up/down regulation 1.6200**

**Spot number 895**

**T-test value 0.045**

**Up/down regulation 1.7002**

**Spot number 1053**

**T-test value 0.008167**

**Up/down regulation 2.3614**

**Spot number 1092**

**T-test value 0.05197**

**Up/down regulation 1.4725**

**Spot number 1096**

**T-test value 0.03611**

**Up/down regulation 1.355**

| 1 | MERRRITSAA | RRSYVSSGEM | MVGGLAPGRR | LGPGTRLSLA | RMPPPLPTRV |
| --- | --- | --- | --- | --- | --- |
| 51 | DFSLAGALNA | GFKETRASER | AEMMELNDRF | ASYIEKVRFL | EQQNKALAAE |
| 101 | LNQLRAKEPT | KLADVYQAEL | RELRLRLDQL | TANSARLEVE | DNLAQDLAT |
| 151 | VRQKLQDETN | LRLEAENNLA | AYRQEADEAT | LARLDLERKI | ESLEEEIRFL |
| 201 | RKIHEEEVRE | LQEQLARQQV | HVELDVAKPD | LTAALKEIRT | YEAMASSNM |
| 251 | HEAEEWYRSK | FADLTDAAAR | NAELLRQAKH | EANDYRRQLQ | LTCDLESLR |
| 301 | GTNESLERQM | REQEERHVRE | AASYQEALAR | LEEEGQSLKD | MARHLQEYQ |
| 351 | DLLNVKLALD | IEIATYRKLL | EGEENRITIP | VQTFSNLQIR | ETSLDTKSVS |
| 401 | EGHLKRNIVV | KTVEMRDGEV | IKESKQEHKD | VM |  |

|  |  | **368** | **417** | **657** | **895** | **1053** | **1092** | **1096** |
| --- | --- | --- | --- | --- | --- | --- | --- | --- |
| **71 - 79** | **R.AEMMELNDR.F** |  |  |  |  |  | **X** |  |
| **71 - 79** | **R.AEMMELNDR.F** Oxidation (M) |  | **X** |  |  |  | **X** | **X** |
| **71 - 79** | **R.AEMMELNDR.F** 2 Oxidation (M) |  | **X** |  | **X** | **X** | **X** | **X** |
| **96 - 105** | **K.ALAAELNQLR.A** |  |  |  |  | **X** | **X** | **X** |
| **112 - 121** | **K.LADVYQAELR.E** | **X** |  |  |  | **X** | **X** | **X** |
| **125 - 136** | **R.LRLDQLTANSAR.L** |  | **X** |  | **X** | **X** | **X** | **X** |
| **127 - 136** | **R.LDQLTANSAR.L** |  | **X** | **X** | **X** |  | **X** | **X** |
| **137 - 152** | **R.LEVERDNLAQDLATVR.Q** |  |  |  |  |  | **X** | **X** |
| **142 - 152** | **R.DNLAQDLATVR.Q** |  | **X** |  |  | **X** | **X** | **X** |
| **153 - 162** | **R.QKLQDETNLR.L** |  |  |  |  |  |  | **X** |
| **153 - 162** | **R.QKLQDETNLR.L** Gln->pyro-Glu (N-term Q) |  | **X** |  |  | **X** |  | **X** |
| **155 - 162** | **K.LQDETNLR.L** |  |  |  |  |  | **X** |  |
| **163 - 173** | **R.LEAENNLAAYR.Q** |  | **X** |  | **X** |  |  | **X** |
| **174 - 183** | **R.QEADEATLAR.L** |  |  |  |  | **X** | **X** | **X** |
| **174 - 183** | **R.QEADEATLAR.L** Gln->pyro-Glu (N-term Q) |  |  |  |  |  | **X** | **X** |
| **189 - 198** | **R.KIESLEEEIR.F** |  | **X** |  | **X** | **X** | **X** | **X** |
| **190 – 198** | **K.IESLEEEIR.F** |  |  |  |  |  |  | **X** |
| **202 – 209** | **R.KIHEEEVR.E** |  |  |  |  | **X** | **X** | **X** |
| **210 - 217** | **R.ELQEQLAR.Q** |  | **X** |  | **X** |  |  |  |
| **218 - 236** | **R.QQVHVELDVAKPDLTAALK.E** Gln->pyro-Glu (N-term Q) |  |  |  |  | **X** |  |  |
| **259 - 270** | **R.SKFADLTDAAAR.N** |  | **X** |  |  | **X** |  |  |
| **261 - 270** | **K.FADLTDAAAR.N** |  | **X** |  | **X** | **X** | **X** |  |
| **301 – 308** | **R.GTNESLER.Q** | **X** |  | **X** |  |  |  |  |
| **301 - 308** | **R.GTNESLER.Q** |  | **X** |  |  |  |  |  |
| **320 - 330** | **R.EAASYQEALAR.L** |  | **X** |  | **X** | **X** | **X** | **X** |
| **331 - 339** | **R.LEEEGQSLK.D** |  | **X** |  |  |  |  |  |
| **331 - 344** | **R.LEEEGQSLKDEMAR.H** |  |  |  |  | **X** | **X** | **X** |
| **331 - 344** | **R.LEEEGQSLKDEMAR.H** Oxidation (M) |  |  |  |  | **X** | **X** | **X** |
| **345 - 356** | **R.HLQEYQDLLNVK.L** |  |  | **X** |  | **X** | **X** | **X** |
| **357 - 367** | **K.LALDIEIATYR.K** |  |  |  |  | **X** | **X** | **X** |
| **368 – 376** | **R.KLLEGEENR.I** |  | **X** |  | **X** | **X** | **X** |  |
| **368 - 376** | **R.KLLEGEENR.I** |  |  |  |  |  |  | **X** |
| **369 - 376** | **K.LLEGEENR.I** |  | **X** |  |  | **X** | **X** | **X** |
| **369 - 390** | **K.LLEGEENRITIPVQTFSNLQIR.E** |  |  |  |  |  |  |  |
| **377 - 390** | **R.ITIPVQTFSNLQIR.E** |  |  |  |  | **X** | **X** | **X** |
| **398 - 406** | **K.SVSEGHLKR.N** | **X** | **X** |  | **X** |  |  |  |
| **412 - 425** | **K.TVEMRDGEVIKESK.Q** |  | **X** |  |  |  |  |  |
| **417 - 425** | **R.DGEVIKESK.Q** | **X** | **X** |  |  |  |  |  |
